# Computation of topological relations with 3-SRM

**DOI:** 10.1038/s41598-026-35579-2

**Published:** 2026-01-23

**Authors:** Nivedita P. Totad, Girish M. Sajjanshettar, Prakash K. Aithal

**Affiliations:** 1 Department of Mathematics, Manipal Institute of Technology, Manipal Academy of Higher Education, Manipal, India; 2 Manipal Institute of Technology, Manipal Academy of Higher Education, Manipal, India

**Keywords:** Topological relations, $9I_A$, $9I_B$, $9I_C$, Ternary relations and 3-SRM, SDG-4, Engineering, Mathematics and computing, Physics

## Abstract

Topological relation models are fundamental to spatial databases and GIS, providing a basis for reasoning about how spatial objects relate. Existing binary frameworks such as RCC-8 and the 9-Intersection Model effectively describe relations between two regions but cannot capture the global structure of configurations involving three spatial entities. To overcome this limitation, we propose a formally defined ternary intersection calculus, the Three-Simple-Region Model (3-SRM), for computing topological relations among three simple regions in 2D space. The model is constructed on the basis of three 3x3 matrices $9I_A$, $9I_B$, and $9I_C$. The configuration of $9I_A$, $9I_B$, and $9I_C$ results in a total of 16 topological relations. The identified topological relations in 2D space among three spatial regions are disjoint, meet, covers, covered-by, equal, contain, inside, overlap, between, in-between, outer, inner, meet-inside, inside-meet, exterior meet, and boundary exterior meet. The model characterizes each triadic relation by rigorously evaluating the emptiness patterns of all interior–boundary–exterior intersections among the three regions, providing a natural extension of traditional binary frameworks while maintaining their fundamental topological semantics.

## Introduction

Spatial information is used to interpret local information in the context of a global scenario. Topological spatial relation models have applications in spatial data modeling, entails spatial queries and spatial reasoning. In geographic information science, region semantics is of primary interest. A spatial database provides information about the relative position, displacement, and geometrical properties of an object.

Objects are complex entities composed of lines, edges and regions in various relations and properties example; shape, size and texture^1^. Space is represented at two levels: the topological level and the geometric level^2^. The way humans perceive and represent space has resulted in cognitive mapping^3,4^. Qualitative models have been suggested^5–7^. The qualitative representation of space is referred to as the qualitativeness of cognitive space^8^. Spatial relations are classified into four categories: set-oriented, topological, metric, and Euclidean^9^. Geometric information system models are based on spatial concept^10^.

Different qualitative topological (and mereotopological) theories have been presented and formalized^11^. Spatial relationships arise on the basis of morphology and position. Positional relations are based on connection and separation. The connection occurs at two levels: topological and geometric. Separations are understood through distance and relevance^12^. A topological relation between two regions becomes uncertain if the regions are imprecise in their location^13,14^.

Qualitative spatial reasoning has been widely discussed in the literature. The properties of physical space have been explored in depth^15^, and an inference scheme based on the composition of relations has been developed. The fundamental spatial relations include proximity, containment, and orientation^16^. A complete classification of topological relation between plane regions using 9IM^17^. A multilevel modeling to describe region -region relations based upon topological invariant^18^. A Voronoi based 9-intersection model for spatial relations is proposed^19^. A complete classification of spatial relations using Voronoi-based nine-intersection model^20^. The Region Connection Calculus (RCC) theory describes the spatiotemporal topological relationships between moving objects^21^. Computation of binary topological relations between complex regions by known topological relations between simple regions^22^. Spatial queries are commonly classified into five categories: topology-based, metric-based, direction-based, hybrid spatial queries, and spatial join queries^23^.

Several topological formalisms for planar regions have been proposed in the literature. Based on point-set topology, the topological relationship between two regions is characterized by the intersections of their interior, boundary, and exterior. The 9-Intersection Model (9IM) and the Region Connection Calculus (RCC)^24^ are well-known qualitative spatial reasoning (QSR) models in GIS.

Topological spatial relations were propounded by Max Egenhofer and Franzosa^25^. These relations are invariant under translation, rotation, and scaling. The definition of topological relationships between two spatial regions in two-dimensional space was presented by Egenhofer in 1990. Based on the intersection of the boundaries and interiors of two regions, the model is known as the 4-Intersection Model (4IM)^26^. Later, with the inclusion of the exteriors of the two regions, a 9-Intersection Model (9IM) was introduced^27^. The 9IM identifies a set of eight topological relations between two simple regions.

The topological relationship between $R^2$ and $Z^2$ was analyzed by^28^. The Region Connection Calculus model, RCC-8, proposed by^24^, defines eight jointly exhaustive and pairwise disjoint relations between two simple regions using the 9-Intersection Model (9IM). Based on spatial intersections, the Dimension-Extended Intersection Model was developed^29^. Building on RCC-8, the RCC-9 model was introduced^30^. All geographical networks can be represented as matrices; however, not all matrices in geography represent networks^31^. Edge contraction decreases structural richness just as region merging reduces the range of topological configurations^32^..Maximal-mean-subtree trees parallel long, stretched spatial regions that offer richer topological interaction patterns^33^. Sub path count reflects a graph’s linear interaction capacity, analogous to how many ways region boundaries can touch or relate^34^.A cactus graph’s single shared vertex is analogous to regions touching at one boundary point in a three-region topological configuration^35^.The matrix method works like a probabilistic version of the 27-relation model, assigning probabilities to each possible region intersection^36^. Diameter-limited subtrees act like small, compact regions whose interactions stay mostly inside or close to the boundary^37^.. RIM (Ray Intersection Model) is a ternary qualitative framework that identifies whether object O is between objects A and B by examining O’s intersections with the ray-defined region connecting A and B. It offers a geometric, ray-based representation of betweenness capturing triadic spatial structure that binary models like RCC-8 or 9-IM are unable to express^38^. It demonstrates that capturing the full topology of spatial scenes requires considering interactions among all regions together, rather than only examining pairwise relationships^39^.

Classical qualitative spatial formalisms such as RCC-8 and the 9-Intersection Model provide robust frameworks for describing topological relations between pairs of spatial regions. However, their binary nature fundamentally limits their ability to represent the global topology of configurations involving three or more objects, as the semantic dependencies that arise in multi-region scenes cannot be recovered from any composition or aggregation of pairwise relations. To address this limitation, we introduce the Three-Simple-Region Model (3-SRM), a formally defined ternary intersection calculus that generalizes the 9IM and RCC ontologies to support explicit three-region reasoning. The 3-SRM framework preserves the interior–boundary–exterior semantics central to RCC while extending expressive power to capture inherently triadic configurations–such as between, in-between, inner, outer, and mixed interior–boundary interactions–that have no representation in binary RCC-8 or 9IM. By offering a minimal, mathematically rigorous, and semantically compatible extension of classical RCC-style topology, 3-SRM provides a principled foundation for qualitative analysis of multi-region spatial scenes in two-dimensional space.

A spatial topological relation model called the Three Simple Region Model (3-SRM) is constructed to identify topological spatial relations among three simple regions. The model is constructed on the basis of $9I_A$, $9I_B$, and $9I_C$. The configurations of $9I_A$, $9I_B$, and $9I_C$ results in a total of 16 topological relations. The topological relations identified in 2D space among the three spatial regions are: disjoint, meet, covers, covered-by, equal, contain, inside, overlap, between, in-between, outer, inner, meet-inside, inside-meet, exterior meet, and boundary exterior meet. Of these 16 topological relations, 8 have binary matrix representations similar to those described by Max Egenhofer using the 9IM for topological relations between two simple regions in 2D space. The analysis is confined to elementary spatial regions, excluding those with internal boundaries or topological complexity.

The structure of this paper is divided into six parts. The first part provides a brief introduction to the literature. The second part covers the relevant topological concepts, region description, limitation of existing models and transition from Binary 9IM to Ternary 3-SRM. The third part describes the Three Simple Region Model (briefly referred to as 3-SRM). The fourth part discusses the topological relations between three simple spatial regions. The fifth part focuses on three simple spatial regions and their specifications. Finally, the conclusions and scope for future work are presented.

## Relevant topological concepts

A topology on *S* is a collection $\tau \in 2^{S}$ satisfying the axioms (1) $\emptyset \in \tau$ and ${S}\in \tau$ (2) $\tau$ is closed under arbitrary union (3) $\tau$ is closed under finite intersection. A topological space is a pair ($\mathcal {S},\tau$), where $\tau$ is a topology on *S*. The elements of $\tau$ are called open sets. The closed set is a subset $\mathcal {A}$ of *S* such that ${S-A}$ is an element of $\tau$.

### Interior, exterior, boundary and closure of a set

In a topological space ${(S,\tau )}$, the interior, exterior, and boundary of a set ${A \subseteq S}$ describe its position within the space. The interior of $\mathcal {A}$, denoted ${A^\circ }$, is defined as the largest open set contained in *A*, that is, ${A^\circ }= \bigcup \{\, U: U \text { is open and } U \subseteq A \,\}$ and a point *x* lies in $A^\circ$ if there exists an open set *U* with $x \in U \subseteq A$. The *exterior* of *A*, written $A^{-}$, is the largest open set disjoint from *A*, equivalently $A^{-}={(S-A)}^\circ$ and a point *x* belongs to $A^{-}$ if there exists an open neighborhood *U* containing *x* such that $U \cap A = \varnothing$. In a topological space $(S,\tau )$, the closure of a set $A \subseteq S$, denoted $\overline{A}$, is the smallest closed set containing *A*. Equivalently, it is the intersection of all closed sets that contain *A*.

$$\begin{aligned} \overline{A} = \bigcap \{\, C: C \text { is closed and } A \subseteq C \,\}. \end{aligned}$$

A point $x \in S$ belongs to $\overline{A}$ if and only if every open neighborhood of *x* intersects *A*. Equivalently $\overline{A} = S - (S-A)^\circ$. In particular $\overline{\varnothing } = \varnothing , \quad \overline{S} = S$ and $\partial A = \overline{A} - A^\circ$.

The boundary of *A*, denoted $\partial A$, is the set of all points whose every open neighborhood meets both *A* and its complement, given by $\partial A = \overline{A} \cap \overline{S - A} \quad \text {or equivalently} \quad \partial A = \overline{A} - A^\circ .$

The sets $A^\circ$, $\partial A$, and $A^{-}$ are mutually disjoint and satisfy S =$A^\circ \cup \partial A \cup A^{-}$ representing points strictly inside, on the edge of, and strictly outside the set *A*.

### Region description

In a topological space S, A is non- empty subset of S, A is said to be regularly closed if A = $\overline{A^\circ }$^40^. A spatial region is defined as a regular closed set with a connected interior. i.e., $A^\circ$ forms a single unbroken open set, has a connected boundary i.e.$\partial (A)$ forms a single continuous edge and it has a connected exterior i.e. $A^{-}$ is a single unbroken region outside A. Given $A \subseteq S$, the *subspace topology* $\tau _A = \{ U \cap A: U \in \tau \}$ allows *A* to inherit all topological properties, including open and closed sets, interior, closure, boundary, and connectedness, from the parent space *S*.

A spatial region without holes, multiple disconnected parts or isolated boundary components is called simple spatial region^21^.. In the literature, regions, objects, or cells are commonly represented using simplicial complexes. In this model simple spatial regions are treated as hexagons without self-intersections.

**Fig. 1:**
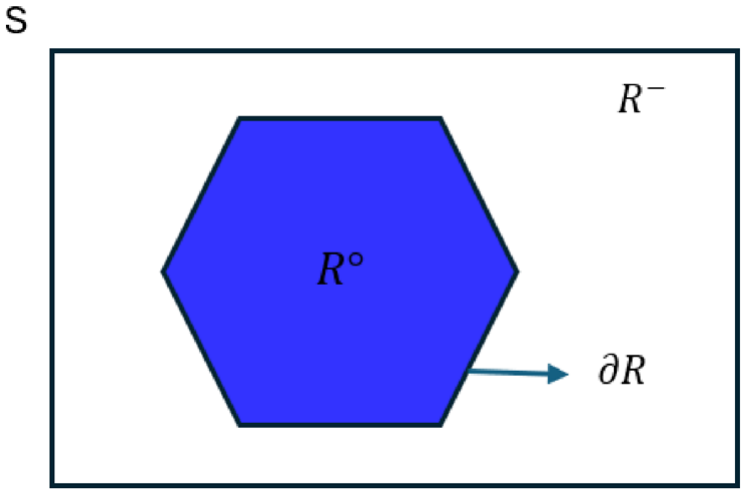
Fundamental topological components.

Figure 1 depicts the fundamental topological components associated with any spatial region *R* its interior, boundary, and exterior within the space *S*.

### Limitations of the existing models

Given three regular closed regions A, B and C in 2-D space the 9 IM and RCC-8 models defined as $M(A,B),\quad M(B,C),\quad M(A,C)$ but these three matrices are independent, global consistency cannot be assured. They cannot express any configuration involving all three regions jointly. Binary 9IM relations form a binary constraint network of the form

$$\begin{aligned} R = \{\, R(A,B),\; R(B,C),\; R(A,C) \,\} \end{aligned}$$

But such networks do not encode higher-order relations because

$$\begin{aligned} R(A,B)\;\wedge \;R(B,C)\;\wedge \;R(A,C)\;\not \Rightarrow \;R(A,B,C) \end{aligned}$$

This is a known limitation in qualitative spatial reasoning i.e. pairwise consistency $\ne$ global consistency. Moreover, binary calculi cannot distinguish between configurations in which triadic structure is essential, such as betweenness, triangular overlap, two-against-one separation, or tripod configurations. This demonstrates the intrinsic insufficiency of binary topological relations for complete reasoning in multi-region settings.

###  Difference in expressive power

Binary 9IM provides relations of the form *R*(*A*, *B*) whereas the ternary model provides relations of the form *R*(*A*, *B*, *C*). The binary to ternary transition changes the underlying logic from binary predicates to a ternary predicate $R: R^3 \rightarrow \{\text {27 relation types}\}.$ This moves the spatial reasoning system from a binary algebra (e.g., RCC-8) to a ternary algebra (3-SRM). This is formally stronger, every ternary relation uniquely determines all binary relations, but not vice versa.

Symbolically $$R^3(A,B,C) \;\Rightarrow \; \big (R^2(A,B),\; R^2(B,C),\; R^2(A,C)\big ),$$

but $\big (R^2(A,B),\; R^2(B,C),\; R^2(A,C)\big ) \not \Rightarrow R^3(A,B,C).$ This non-equivalence is exactly why the ternary model is necessary.

###  Transition from binary 9IM to ternary 3-SRM

In this section, we present a formal transition from the classical binary 9IM framework to a ternary intersection calculus, which forms the basis of our 3-SRM triadic model. This extension preserves local topological semantics while enabling representation of global configurations that cannot be captured by any combination of binary relations. Instead of the binary intersection $I(A,B) = A^\circ \cap B^\circ ,$ we define the ternary intersection$I(A,B,C) = A^\circ \cap B^\circ \cap C^\circ ,$ and similarly for all combinations of interior, boundary, and exterior. The binary 9IM partitions the plane into 9 parts, whereas the ternary model partitions it into 27 parts. We structured the space of 27 realizable ternary relations into eight topologically consistent families Overlapping, Nested, Separated, Touching, Chain-like, Sandwich-like, Between, and Enclosure.

## Three simple region model (briefly 3-SRM)

### Formal construction of 3-SRM

Consider three simple spatial regions A, B, and C in a two-dimensional space with co-dimension zero. To identify the spatial connections among these regions, a model is developed based on the interiors, boundaries, and exteriors of the regions and their intersections.

The model defines T(A, B, C) $=$ pattern of nonempty triple intersections $X_A \,\cap \, X_B \,\cap \, X_C,\quad X \in \{\circ ,\, \partial ,\, -\}$

Model is designed on the bases of $\{\, A^{\circ },\; \partial A,\; A^{-} \,\}$, $\{\, B^{\circ },\; \partial B,\; B ^{-} \,\}$ and $\{\, C^{\circ },\; \partial c,\; C^{-} \,\}$ then the space is partitioned into $(A^{\circ },\, \partial A,\, A^{-}) \times (B^{\circ },\, \partial B,\, B^{-}) \times (C^{\circ },\, \partial C,\, C^{-})$. Each of the 27 cubes of this 3$\times 3\times$3 partition can be empty and nonempty and these patterns determine the triadic relation. Each cell is $C_{ijk} = X_{A_i} \;\cap \; X_{B_j} \;\cap \; X_{C_k}$, where i, j, k $=$ 1,2,3 patterns of non-empty cubes define one of the 27 canonical triadic relations. In 3-SRM, $X_{A_1}$ $=$ $9I_A$, $X_{B_1}$ $=$ $9I_B$ and $X_{C_1}$ $=$ $9I_C$.

The 27 relations are organized into three 3$\times$3 matrices, denoted by $9 I_A$, $9 I_B$, and $9 I_C$, each representing a distinct topological relation among the regions. Collectively, these matrices constitute the Three-Simple Region Model (3-SRM). The matrix forms of $9 I_A$, $9 I_B$ and $9 I_C$ are presented below :

$$\begin{aligned} & \begin{array}{ccc} 9I_A= \begin{bmatrix} A^\circ \cap (B^\circ \cap C^\circ ) & \partial (A) \cap (B^\circ \cap C^\circ ) & A^{-} \cap (B^\circ \cap C^\circ ) \\ A^\circ \cap (\partial (B) \cap (\partial (C)) & \partial (A) \cap (\partial (B) \cap \partial (C)) & A^{-} \cap (\partial (B)\cap \partial (C)) \\ A^\circ \cap (B^{-} \cap C^{-}) & \partial (A) \cap (B^{-}\cap C^{-}) & A^{-} \cap (B^{-} \cap C^{-}) \end{bmatrix} \end{array}\\ & \begin{array}{ccc} 9I_B= \begin{bmatrix} B^\circ \cap (A^\circ \cap C^\circ ) & \partial (B) \cap (A^\circ \cap C^\circ ) & B^{-} \cap (A^\circ \cap C^\circ ) \\ B^\circ \cap (\partial (A) \cap (\partial (C)) & \partial (B) \cap (\partial (A) \cap \partial (C)) & B^{-} \cap (\partial (A)\cap \partial (C)) \\ B^\circ \cap (A^{-} \cap C^{-}) & \partial (B) \cap (A^{-} \cap C^{-}) & B^{-}\cap (A^{-} \cap C^{-}) \end{bmatrix} \end{array}\\ & \begin{array}{ccc} 9I_C= \begin{bmatrix} C^\circ \cap (A^\circ \cap B^\circ ) & \partial (C) \cap (A^\circ \cap B^\circ ) & C^{-} \cap (A^\circ \cap B^\circ ) \\ C^\circ \cap (\partial (A) \cap (\partial (B)) & \partial (C) \cap (\partial (A) \cap \partial (B)) & C^{-} \cap (\partial (A)\cap \partial (B)) \\ C^\circ \cap ( A^{-} \cap B^{-}) & \partial (C) \cap ( A^{-}\cap B^{-}) & C^{-}\cap ( A^{-} \cap B^{-}) \end{bmatrix} \end{array} \end{aligned}$$

The binary computation adopted in this model is derived from the right-hand side of the distributive property, without enforcing an equality constraint. For instance, $A^\circ \cap (B^\circ \cap C^\circ ) = (A^\circ \cap B^\circ ) \cap (A^\circ \cap C^\circ )$ and analogous expressions apply to all combinations of interior, boundary, and exterior components. Each resulting intersection is represented as a binary value–0 for an empty intersection and 1 for a non-empty one–providing a uniform encoding of all triadic intersection patterns.

## Topological relations between three simple spatial regions

Based on the calculations of $9I_A$, $9I_B$, and $9I_C$ and their configurations, 16 topological relations are defined. Each of $9I_A$, $9I_B$, and $9I_C$ corresponds to at least one of these 16 topological relations.

### Definition 1

If the interior and boundary of one region intersect with the exterior of the other two regions, and the exterior of the same region intersects with the interior, boundary, and exterior of the other two regions, then the region has a *disjoint* relation.

### Definition 2

If the boundary and exterior of one region intersects boundaries and exteriors of other two regions then the region has a *meet* relation.

### Definition 3

If the interior, boundary, and exterior of one region intersect the interior, boundary, and exterior of the other two regions, then the region has the *equal* relation.

### Definition 4

If the interior of one region intersects with the interior, boundary, and exterior of the other two regions, and its boundary and exterior intersect only with the exterior of the other two regions, then the region has the *inside* relation.

### Definition 5

If the interior and boundary of a region intersect with the interiors of the other two regions, and the exterior of the same region intersects with the interiors, boundaries, and exteriors of the other two regions, then the region has the *contain* relation.

### Definition 6

If the interior and boundary of a region intersect with the interior of the other two regions, and the exterior of the same region intersects with the interior, boundary, and exterior of the other two regions, and additionally, the boundary of the same region intersects with the boundaries of the other two regions, then the region has the *covers* relation.

### Definition 7

If the interior of one region intersects with the interior, boundary, and exterior of the other two regions, and its boundary and exterior intersect with the exterior of the other two regions – and its boundary also intersects with the boundaries of the other two regions – then the region has the *coveredby* relation.

### Definition 8

If all three regions intersect in their interior, boundary, and exterior parts, then the regions exhibit the *overlap* relation.

### Definition 9

If all three spatial regions intersect through their interiors and exteriors, then the relation is called *between*.

### Definition 10

If all three spatial regions intersects with their interior, boundary and exterior then it is $in-between$ relation.

### Definition 11

If the interior, boundary, and exterior of one region intersect only with the exteriors of the other two regions, then the region has an *outer* relation.

### Definition 12

If the exterior of one region intersects with the interior, boundary, and exterior of the other two regions, then the region has an *inner* relation.

### Definition 13

If the exterior of one region intersects with the interior, boundary, and exterior of the other two regions, and its boundary also intersects the boundaries of the other two regions, then the region exhibits the *meet*–*inside* relation.

### Definition 14

If the interior, boundary, and exterior of one region intersect with the exterior of the other two regions, and its boundary also intersects with the boundaries of the other two regions, then the relation is called $inside\text {-}meet$.

### Definition 15

The relation is $exterior\text {-}meet$ if the exterior of one region intersects with the exteriors of the other two regions.

### Definition 16

If the boundary of one region intersects with the boundaries of the other two regions, and its exterior intersects with the exteriors of the other two regions, then the relation is called *boundary*-*exterior*-*meet*.

Within the 3-SRM, the matrices $9I_A$, $9I_B$, and $9I_C$ encode the interaction of each region with the pair formed by the remaining two. Their configurations are not constrained to be uniform: all three may coincide, they may differ entirely, or two may align while the third diverges. These admissible combinations define the 16 realizable three-region topological relations, and each of the matrices $9I_A$, $9I_B$, and $9I_C$ maps onto at least one of these relations.

The 16 topological relations among three simple regions, derived from 3-SRM, are listed in Table 1. and toplogical spatial relation and binary matrices is depicted in Table 2

**Table 1:** The 16 topological relations among three simple regions, derived from 3-SRM.

Topological spatial relation	Binary matrices
Disjoint	$$\begin{bmatrix} 0 & 0 & 1\\ 0 & 0 & 1\\ 1 & 1 & 1 \end{bmatrix}$$
Meet	$$\begin{bmatrix} 0 & 0 & 1\\ 0 & 1 & 1\\ 1 & 1 & 1 \end{bmatrix}$$
Equal	$$\begin{bmatrix} 1 & 0 & 0\\ 0 & 1 & 0\\ 0 & 0 & 1 \end{bmatrix}$$
Contain	$$\begin{bmatrix} 1 & 1 & 1\\ 0 & 0 & 1\\ 0 & 0 & 1 \end{bmatrix}$$
Cover	$$\begin{bmatrix} 1 & 1 & 1\\ 0 & 1 & 1\\ 0 & 0 & 1 \end{bmatrix}$$
Inside	$$\begin{bmatrix} 1 & 0 & 0\\ 1 & 0 & 0\\ 1 & 1 & 1 \end{bmatrix}$$
Covered-by	$$\begin{bmatrix} 1 & 0 & 0\\ 1 & 1 & 0\\ 1 & 1 & 1 \end{bmatrix}$$
Overlap	$$\begin{bmatrix} 1 & 1 & 1\\ 1 & 1 & 1\\ 1 & 1 & 1 \end{bmatrix}$$
Inner	$$\begin{bmatrix}0 & 0 & 1 \\ 0 & 0 & 1 \\ 0 & 0 & 1\end{bmatrix}$$
Outer	$$\begin{bmatrix}0 & 0 & 0 \\ 0 & 0 & 0 \\ 1 & 1 & 1\end{bmatrix}$$
Between	$$\begin{bmatrix} 1 & 0 & 0\\ 0 & 0 & 0\\ 0 & 0 & 1 \end{bmatrix}$$

**Table 2:** The topological spatial relation and binary matrices.

Topological spatial relation	Binary matrices
In-between	$$\begin{bmatrix} 1 & 0& 0\\ 0 & 1 & 0\\ 0 & 0 & 1 \end{bmatrix}$$
Meet inside	$$\begin{bmatrix} 0 & 0 & 1\\ 0 & 1 & 1\\ 0 & 0 & 1 \end{bmatrix}$$
Inside meet	$$\begin{bmatrix} 0 & 0 & 0\\ 0 & 1 & 0\\ 1 & 1 & 1 \end{bmatrix}$$
Exterior meet	$$\begin{bmatrix} 0 & 0 & 0\\ 0 & 0 & 0\\ 0 & 0 & 1 \end{bmatrix}$$
Boundary-exterior-meet	$$\begin{bmatrix} 0 & 0 & 0\\ 0 & 1 & 0\\ 0 & 0 & 1 \end{bmatrix}$$

### Topological connections

Based on the interior, boundary, and exterior of the spatial regions A, B, and C, the connection between these regions is characterized by the matrices $9I_A$, $9I_B$, and $9I_C$. A relation matrix R(A,B,C) provides a unified characterization of $9I_A$, $9I_B$, and $9I_C$. In R(A,B,C), each entry denoted by * can take a value of 0 or 1.

**Case 1**: Boundary of one region is disjoint from interior of other two regions. then, $\partial (A) \cap (B^\circ \cap C^\circ ) = 0$ then,

$$\begin{aligned} R(A,B,C) = \begin{bmatrix} * & 0 & * \\ * & * & * \\ * & * & * \end{bmatrix}\end{aligned}$$

**Case 2**: One region interior intersects with boundary of other two regions. i.e., $A^\circ \cap (\partial (B) \cap \partial (C)) = 1$

$$\begin{aligned} R(A,B,C) = \begin{bmatrix} * & * & * \\ 1 & * & * \\ * & * & * \end{bmatrix}\end{aligned}$$

**Case 3**: Boundary of one region intersects at least one interior of the remaining two regions or if two of the regions follows disjoint or meet or covered-by relation.i.e., $\partial (A)\cap (B^\circ \cap C^\circ ) =0$ then,

$$\begin{aligned} R(A,B,C) = \begin{bmatrix} * & 0 & * \\ * & * & * \\ * & * & * \end{bmatrix}\end{aligned}$$

**Case 4**: Boundary of one region is subset of interior of other two regions. i.e., $\partial (A)\cap (B^\circ \cap C^\circ ) = 1$ then $\partial (A)\cap (\partial (B) \cap \partial (C)) = 0$ and $\partial (A)\cap (B^{-} \cap C^{-}) = 0$ so that,

$$\begin{aligned} R(A,B,C) = \begin{bmatrix} * & 1 & * \\ * & * & * \\ * & * & * \end{bmatrix} \bigvee \begin{bmatrix} * & 0 & * \\ * & 0 & * \\ * & 0 & * \end{bmatrix} \bigvee \begin{bmatrix} * & 0 & * \\ * & 0 & * \\ * & 1 & * \end{bmatrix} \end{aligned}$$

**Case 5**: Boundary of one region is subset of remaining two regions. i.e.,

$$\begin{aligned} \partial (A) \subset B, C \Rightarrow \partial (A)\subset (\partial (B) \cup B^\circ ), \quad \partial (A)\subset (\partial (C) \cup C^\circ ) \end{aligned}$$

then,

$$\begin{aligned} R(A,B,C) = \begin{bmatrix} * & 1 & * \\ * & 1 & * \\ * & 0 & * \end{bmatrix} \bigvee \begin{bmatrix} * & 0 & * \\ * & 1 & * \\ * & 0 & * \end{bmatrix} \bigvee \begin{bmatrix} * & 0 & * \\ * & 1 & * \\ * & 1 & * \end{bmatrix}\end{aligned}$$

**Case 6**: If the boundary of all the regions coinside i.e. $\partial (A) \cap (\partial (B) \cap \partial (C))$ = 1 then,

$$\begin{aligned} R(A,B,C) = \begin{bmatrix} * & 0 & * \\ 0 & 1 & 0 \\ * & 0 & * \end{bmatrix} \bigvee \begin{bmatrix} * & 1 & * \\ 0 & 1 & 1 \\ * & 0 & * \end{bmatrix} \bigvee \begin{bmatrix} * & 0 & * \\ 1 & 1 & 0 \\ * & 1 & * \end{bmatrix} \begin{bmatrix} * & 0 & * \\ 0 & 1 & 0 \\ * & 1 & * \end{bmatrix} \bigvee \begin{bmatrix} * & 0 & * \\ 0 & 1 & 1 \\ * & 0 & * \end{bmatrix} \bigvee \begin{bmatrix} * & 0 & * \\ 0 & 1 & 1 \\ * & 1 & * \end{bmatrix} \end{aligned}$$

###  Regions and conditions

**Condition 1**: The exterior of the regions intersects with each other.

$$\begin{aligned} R(A,B,C) \ne \begin{bmatrix} * & * & * \\ * & * & * \\ * & * & 0 \end{bmatrix} \end{aligned}$$

**Condition 2**: If the interior of one region does not intersect with the interiors of the other two regions, then its interior intersects with the exterior and interior of the other two regions.

For example, If $A^\circ \cap (B^\circ \cap C^\circ ) = 0$ then $A^\circ \cap (\overline{B} \cup \overline{C})$= 1 and $A^{-} \cap (B^\circ \cap C^\circ ) = 1$

$$\begin{aligned} R(A,B,C) \ne \begin{bmatrix} 1 & * & 0 \\ * & * & * \\ 0 & * & * \end{bmatrix} \end{aligned}$$

**Condition 3**: If the interior of one region is inside the exterior of the other two regions, then the boundary of the same region is also in the exterior of the other two regions.

For example, In $9I_A$, $A^\circ \subset (B^{-} \cap C^{-})$ then $\partial (A) \subset (B^{-} \cap C^{-}).$ similarly in $9I_B$, $B^\circ \subset (A^{-} \cap C^{-})$ then $\partial (B) \subset (A^{-} \cap C^{-})$ and in $9I_C$, $C^\circ \subset (A^{-} \cap B^{-})$ then $\partial (C) \subset (A^{-} \cap B^{-})$

$$\begin{aligned} R(A,B,C) \ne \begin{bmatrix} * & * & * \\ * & * & * \\ 0 & 0 & * \end{bmatrix} \bigvee \begin{bmatrix} * & * & * \\ * & * & * \\ 1 & 1 & * \end{bmatrix}\end{aligned}$$

**Condition 4**: If the interior of one region intersects with the boundaries of the other two regions, then the interior of the same region is also in the exterior of the other two regions.

For example, In $9I_A$, if $A^\circ \cap (\partial (B) \cap \partial (C))$ = 1 then $A^\circ \cap (B^{-} \cap C^{-})$ = 1

$$\begin{aligned} R(A,B,C) \ne \begin{bmatrix} * & * & * \\ 0 & * & * \\ 0 & * & * \end{bmatrix} \bigvee \begin{bmatrix} * & * & * \\ 1 & * & * \\ 1 & * & * \end{bmatrix}\end{aligned}$$

**Condition 5**: The boundary of one region intersects with either the interior, exterior, or boundary of the other two regions.

For example, $\partial (A) \cap (B^\circ \cap C^\circ )$= 1 or $\partial (A) \cap (\partial (B) \cap \partial (C))$=1 or $\partial (A) \cap (B^{-} \cap C^{-})=1$

$$\begin{aligned} R(A,B,C) \ne \begin{bmatrix} * & 0 & * \\ * & 0 & * \\ * & 0 & * \end{bmatrix} \end{aligned}$$

**Condition 6**: If the interior of one region is disjoint from the interiors of the other two regions, then the boundary of that region is also disjoint from the interiors of the other two regions.

For example, if $A^\circ \cap (B^\circ \cap C^\circ )$ = 0 then $\partial (A) \cap (B^\circ \cap C^\circ )$ = 0 and $A^\circ \cap (\partial (B) \cap \partial (C))$ = 0

$$\begin{aligned} R(A,B,C) \ne \begin{bmatrix} 1 & 1 & * \\ 1 & * & * \\ * & * & * \end{bmatrix} \end{aligned}$$

**Condition 7**: If the interior of one region intersects with the interior and exterior of the other two regions, then it also intersects with their boundaries.

For example, If $A^\circ \cap (B^\circ \cap C^\circ )$ = 1 and $A^\circ \cap (B^{-} \cap C^{-}) = 1$ then $A^\circ \cap (\partial (B) \cap \partial (C)) = 1$

or $A^\circ \cap (B^\circ \cap C^\circ )$ = 1 and $A^{-} \cap (B^\circ \cap C^\circ ) = 1$ then $\partial (A) \cap (B^\circ \cap C^\circ ) = 1$

$$\begin{aligned} R(A,B,C) \ne \begin{bmatrix} 0 & * & * \\ 0 & * & * \\ 0 & * & * \end{bmatrix} \bigvee \begin{bmatrix} * & 0 & * \\ * & 0 & * \\ * & 0 & * \end{bmatrix} \end{aligned}$$

**Condition 8**: If the boundary of one region does not intersect with the boundaries of the other two regions, then the boundary of that region intersects with the exteriors of the other two regions.

For example, If $\partial (A) \cap (\partial (B) \cap \partial (C)) = 0$ then $\partial (A) \cap (B^{-} \cap C^{-})=1$

$$\begin{aligned} R(A,B,C) \ne \begin{bmatrix} * & * & * \\ * & 1 & * \\ * & 0 & * \end{bmatrix} \bigvee \begin{bmatrix} * & * & * \\ * & 1 & * \\ * & 1 & * \end{bmatrix} \end{aligned}$$

**Condition 9**: If the interior of one region is disjoint from the other two regions, then that region lies either on the boundary or in the exterior of the other regions.

For example, if $A^\circ \cap (B^\circ \cap C^\circ )$ = 0 then $\partial (A) \cap (B^{-} \cap C^{-}) = 1$ or $A^{-} \cap (\partial (B) \cap \partial (C)) = 1$

$$\begin{aligned} R(A,B,C) \ne \begin{bmatrix} 1 & * & * \\ * & * & 0 \\ * & 0 & * \end{bmatrix} \end{aligned}$$

**Condition 10**: If the boundary of one region intersects with the interiors of the other two regions, and the interior of that region intersects with the boundaries of the other two, then the boundary of that region also intersects with the boundaries of the other two regions.

For example, if $\partial (A) \cap (B^\circ \cap C^\circ ) = 1$ and $A^\circ \cap (\partial (B) \cap \partial (C)) = 1$ then $\partial (A) \cap (\partial (B) \cap \partial (C)) = 1$

$$\begin{aligned} R(A,B,C) \ne \begin{bmatrix} * & 0 & * \\ 0 & 0 & * \\ * & * & * \end{bmatrix} \end{aligned}$$

**Condition 11**: If the interior of one region intersects with the exteriors of the other two regions, then the boundary of that region intersects with the exteriors of the other two regions.

For example,if $A^\circ \cap (B^{-} \cap C^{-})=1$ then $\partial (A) \cap (B^{-} \cap C^{-}) =1$

$$\begin{aligned} R(A,B,C) \ne \begin{bmatrix} * & * & * \\ * & * & * \\ 0 & 0 & * \end{bmatrix}\end{aligned}$$

**Condition 12**: If the boundary of one region intersects with the interiors of the other two regions, then the exterior of the same region also intersects with the interiors of the other two regions.

For example, if $\partial (A) \cap (B^\circ \cap C^\circ )$ = 1 then $A^{-}\cap (B^\circ \cap C^\circ )=1$

$$\begin{aligned} R(A,B,C) \ne \begin{bmatrix} * & 0 & 0 \\ * & * & * \\ * & * & * \end{bmatrix}\end{aligned}$$

**Condition 13**: If the interior of one region intersects with the boundaries of the other two regions, then the exterior and boundary of that region do not intersect with the boundaries of the other two regions.

For example, if $A^\circ \cap (\partial (B) \cap \partial (C))$ = 1 then $A^{-}\cap (\partial (B) \cap \partial (C))$ = 0 and $\partial (A)\cap (\partial (B)\cap \partial (C))$=0

$$\begin{aligned} R(A,B,C) \ne \begin{bmatrix} * & * & * \\ 0 & 1 & 1 \\ * & * & * \end{bmatrix} \bigvee \begin{bmatrix} * & * & * \\ 1 & 1 & 1 \\ * & * & * \end{bmatrix} \bigvee \begin{bmatrix} * & * & * \\ 1 & 1 & 0 \\ * & * & * \end{bmatrix} \end{aligned}$$

**Condition 14**: Case 1 : If the boundary of one region intersects with the interiors of the other two regions, then the boundary of the same region also intersects with the boundaries of the other two regions and does not intersect with their exteriors.

For example if $\partial (A) \cap (B^\circ \cap C^\circ )$=1 then $\partial (A) \cap (B^{-}\cap C^{-})$=0 and $\partial (A) \cap (\partial (B)\cap \partial (C))$=1

$$\begin{aligned} R(A,B,C) \ne \begin{bmatrix} * & 0 & * \\ * & 0 & * \\ * & 1 & * \end{bmatrix} \end{aligned}$$

Case 2: If the boundary of one region intersects with the interiors of the other two regions, then the boundary of that region does not intersect with the boundaries and exteriors of the other two regions.

For example, if $\partial (A) \cap (B^\circ \cap C^\circ )$=1 then $\partial (A) \cap (B^{-}\cap C^{-})$=0 and $\partial (A) \cap (\partial (B)\cap \partial (C))$=0

$$\begin{aligned} R(A,B,C) \ne \begin{bmatrix} * & 0 & * \\ * & 1 & * \\ * & 1 & * \end{bmatrix} \end{aligned}$$

### Spatial regions with interior, closure and boundary relations

#### Proposition 1

*Let B,C be a two spatial region in X. If* $B^\circ \cap C^\circ \ne \emptyset$ *and* $\partial (B) \cap \partial (C) = \emptyset$ *then either B is subset of C or vice versa.*

#### Proof

Suppose $B^\circ \cap C^\circ \ne \emptyset$ and $\partial (B) \cap \partial (C) = \emptyset$ then $\partial (B) \subset X-\partial (C)$ by Proposition 3.4^25^ $\partial (B) \subset C^\circ \cup (X-{\bar{C}})$ consequently $\partial (B) \subset C^\circ$ hence it follows B is a subset of C. $\square$

#### Proposition 2

*Let A, B, C be spatial region in X. If* $A^\circ \cap (B^\circ \cap C^\circ ) \ne \emptyset$ *and* $\partial (A)\cap (\partial (B)\cap \partial (C)) =\emptyset$ *provided* $(\partial (A)\cap \partial (B))=\emptyset$, $(\partial (A)\cap \partial (C))=\emptyset$ *then B and C are subsets of A. Also B is subset of C*.

#### Proof

Suppose $A^\circ \cap (B^\circ \cap C^\circ ) \ne \emptyset$ holds then $(A^\circ \cap B^\circ ) \ne \emptyset$, $(A^\circ \cap C^\circ ) \ne \emptyset$ since

$\partial (A) \cap (\partial (B) \cap \partial (C)) = \emptyset$ and $(\partial (A)\cap \partial (B))=\emptyset$, $(\partial (A)\cap \partial (C))=\emptyset$ which implies

$\partial (B) \subset X-\partial (A)$, $\partial (C) \subset X-\partial (A)$ further $\partial (B) \subset A^\circ \cup (X-\bar{A})$, $\partial (C) \subset A^\circ \cup (X-\bar{A})$ by Proposition 3.4^25^ $\partial (B) \subset A^\circ$, $\partial (C) \subset A^\circ$ so $\partial (B), \partial (C) \subset A^\circ$ hence, B and C are subsets of A. by hypothesis $B^\circ \cap C^\circ \ne \emptyset$, $\partial (B) \cap \partial (C) = \emptyset$ by Proposition 1, B is subset of C. $\square$

#### Proposition 3

*Let A, B, C are spatial regions in X. If* $A^\circ \cap (B^\circ \cap C^\circ ) \ne \emptyset$ *and* $A^\circ \cap (\partial (B)\cap \partial (C)) =\emptyset$ *then region A is inside region B and C.*

#### Proof

Suppose $A^\circ \cap (B^\circ \cap C^\circ ) \ne \emptyset$ holds then $(A^\circ \cap B^\circ ) \ne \emptyset$, $(A^\circ \cap C^\circ ) \ne \emptyset$ also

$A^\circ \cap (\partial (B) \cap \partial (C)) = \emptyset$ which gives $(A^\circ \cap \partial (B))=\emptyset$, $(A^\circ \cap \partial (C))=\emptyset$ Proposition 5.5^25^ A $\subset$ B,

A $\subset$ C Hence A is inside B and C. By hypothesis $B^\circ \cap C^\circ \ne \emptyset$, $\partial (B) \cap \partial (C) = \emptyset$ by Proposition 1 B is subset of C. $\square$

#### Proposition 4

*Spatial regions A, B, C are equal i.e, A=B=C. if (i)* $A^\circ \cap (B^\circ \cap C^\circ )\ne \emptyset$

*(ii)* $A^\circ \cap (\partial (B) \cap \partial (C)) = \emptyset$ *and (iii)* $\partial (A)\cap (B^\circ \cap C^\circ ) = \emptyset$

#### Proof

By hypothesis (i), (ii), Proposition 3 and Proposition 1 yields $A \subset B \subset C$.

Also by hypothesis (i) $(A^\circ \cap B^\circ ) \ne \emptyset$, $(A^\circ \cap C^\circ ) \ne \emptyset$ and by hypothesis (iii) $\partial (A) \cap B^\circ =\emptyset , \partial (A) \cap C^\circ =\emptyset$ combining above and using Proposition 5.5^25^, $C \subset B \subset A$. Hence from above A=B=C. $\square$

#### Proposition 5

*If A, B, C are spatial regions in X and if* $C\subset B \subset A$ *then* $C\subset B^\circ \subset A^\circ$

#### Proof

Proof follows by Proposition 4 and 3.2^25^. $\square$

## Three simple spatial regions and their specifications

**Fig. 2:**
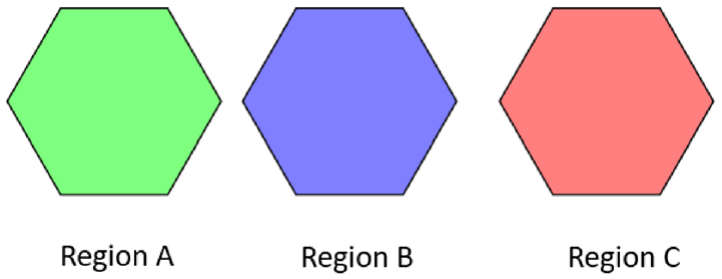
Spatial regions.

The spatial regions A, B, and C are green, blue, and red, respectively as depicted in Fig. 2

By analyzing the empty and non-empty intersections among the interior, boundary, and exterior of three spatial regions, and based on the configurations of $9I_A$, $9I_B$, and $9I_C$, the Three Simple Region Model (3-SRM) yields 45 distinct specifications. Each specification is geometrically unique. The spatial configurations and their specifications are depicted as per Table 3 :

**Table 3:** Figure-wise relation specification.

Figure number	Relation specification
Fig. 3	Disjoint
Fig. 4	Meet
Fig. 5	Equal
Fig. 6	Overlap
Fig. 7	Inside, between, and contains
Fig. 8	Covered by, in-between, and covers
Fig. 9	Disjoint and overlap
Fig. 10	Meet and overlap
Fig. 11	Equal and overlap
Fig. 12	Covers, covered-by, and overlap
Fig. 13	Inside, overlap, and contain
Fig. 14	Inside, inner, and disjoint
Fig. 15	Meet, inner, and inside
Fig. 16	Outer, inner, and disjoint
Fig. 17	Covered-by, contains, and inside
Fig. 18	Inside and contain
Fig. 19	Inside, inner, and inner
Fig. 20	Inside and contains
Fig. 21	Inside and between
Fig. 22	Between and contains
Fig. 23	Inside, contain, and cover
Fig. 24	Inside, in-between, and contains
Fig. 25	Covered-by and inner
Fig. 26	Covered-by and meet-inside
Fig. 27	Covered-by and covers
Fig. 28	Disjoint and meet
Fig. 29	Overlaps and equal
Fig. 30	Inside-meet, meet-inside, and meet
Fig. 31	Equal and covered-by
Fig. 32	Equal and covers
Fig. 33	Inner, inside-meet, and disjoint
Fig. 34	Disjoint, outer, and inner
Fig. 35	Inside, inner, and meet-inside
Fig. 36	Covered-by and covers
Fig. 37	Covered-by, inner, and disjoint
Fig. 38	Inside-meet, inner, and disjoint
Fig. 39	Covered-by, inside-meet, and meet
Fig. 40	Meet and boundary exterior meet
Fig. 41	Exterior-meet and disjoint

**Fig. 3:**
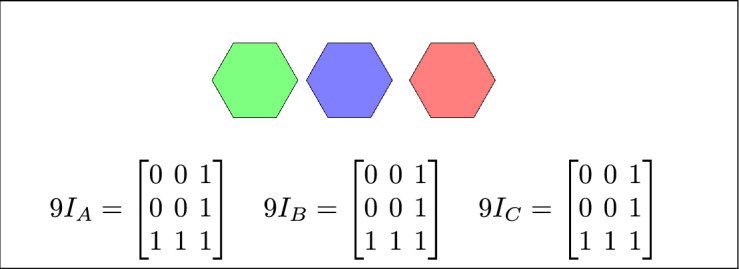
Regions A, B and C are disjoint.

**Fig. 4:**
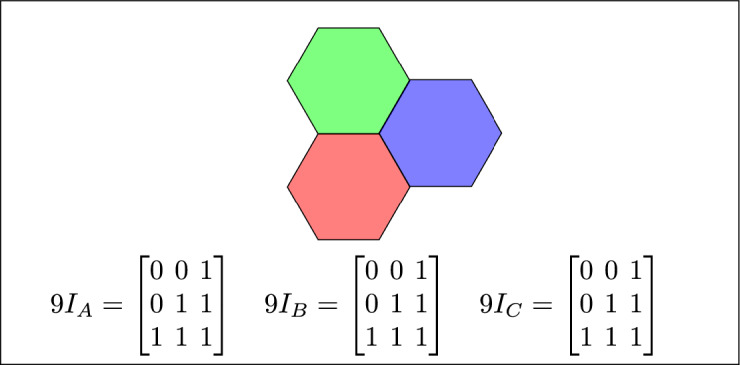
Regions A, B and C meet each other.

**Fig. 5:**
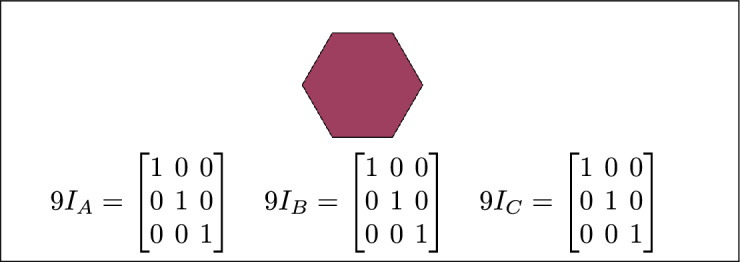
Regions A, B and C are equal.

**Fig. 6:**
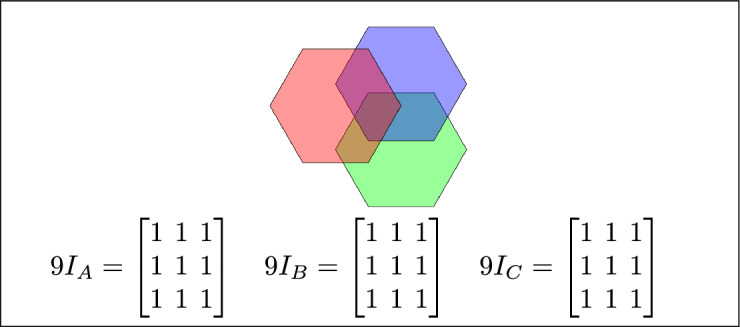
Regions A, B, and C overlap.

**Fig. 7:**
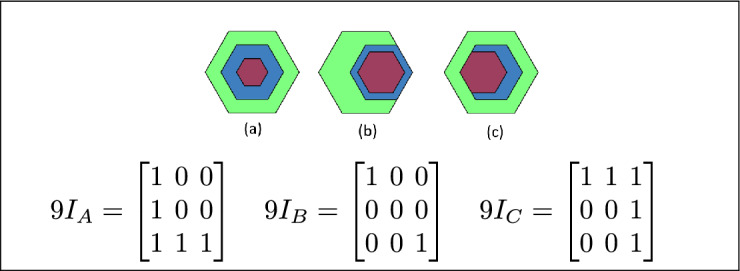
(**a**) B and C are inside A (**b**) B lies between C and A (**c**) A contains both B and C.

**Fig. 8:**
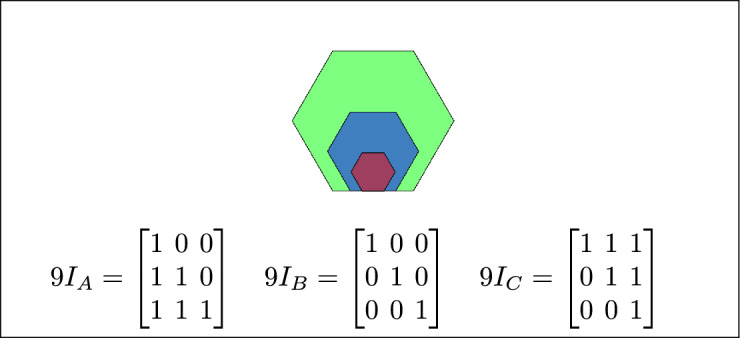
C is covered by B and A, B is in-between C and A, and A covers both B and C.

**Fig. 9:**
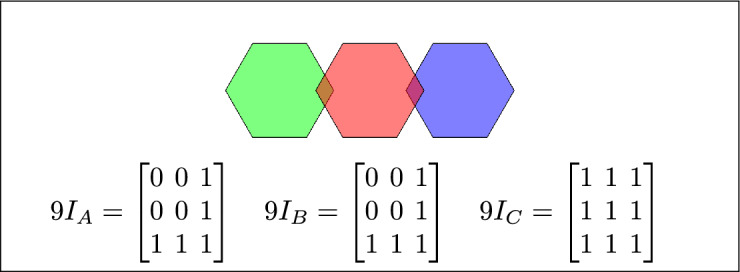
A is disjoint from B, and C overlaps with both A and B.

**Fig. 10:**
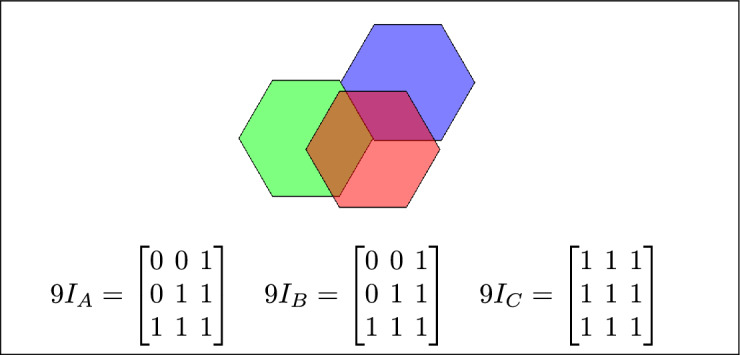
A meets B, and C overlaps with both A and C.

**Fig. 11:**
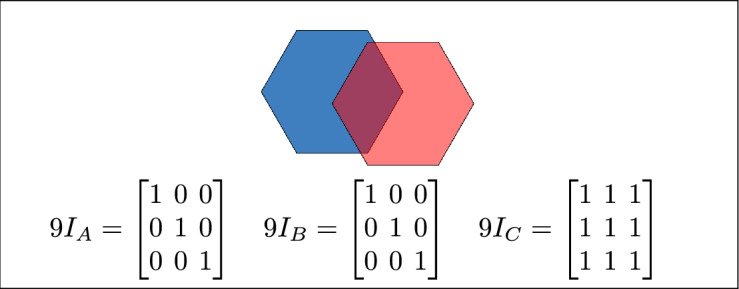
A and B are equal, and C overlaps with both A and B.

**Fig. 12:**
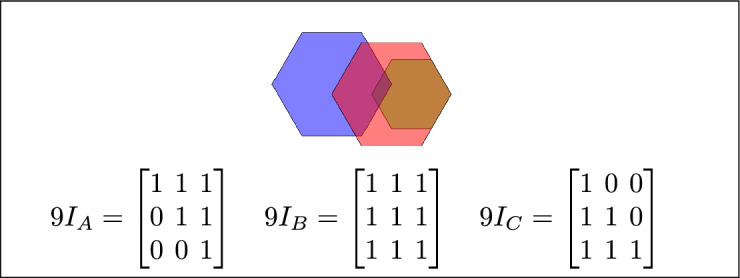
C covers A, B overlaps with both C and A, and A is covered by C.

**Fig. 13:**
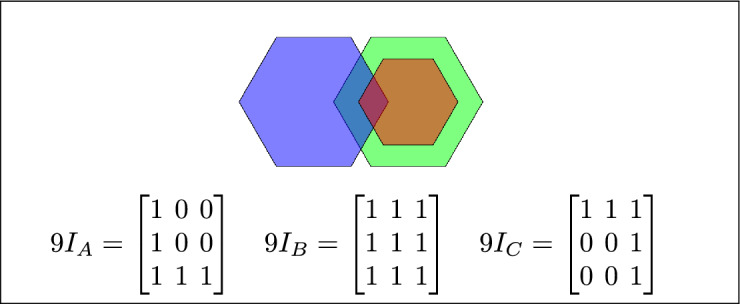
C is inside A, B overlaps with A and C, and A contains C.

**Fig. 14:**
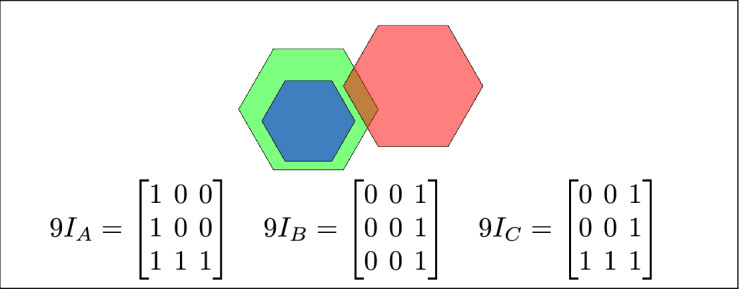
B is inside A, B is the inner region, and C is disjoint from B.

**Fig. 15:**
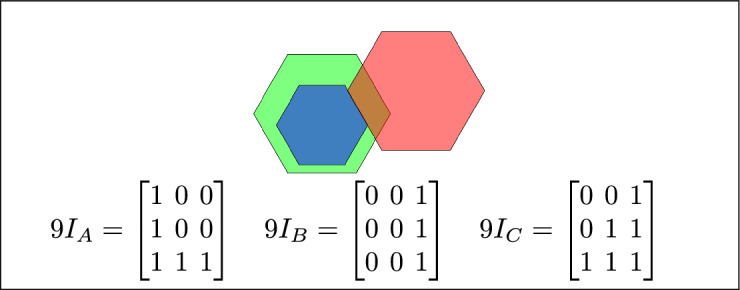
B is inside A, B is the inner region, and region C meets B.

**Fig. 16:**
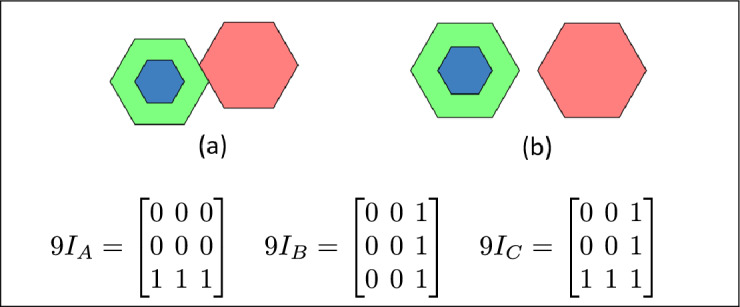
In (**a**) and (**b**) A is the outer region, B is the inner region, and C is disjoint from B.

**Fig. 17:**
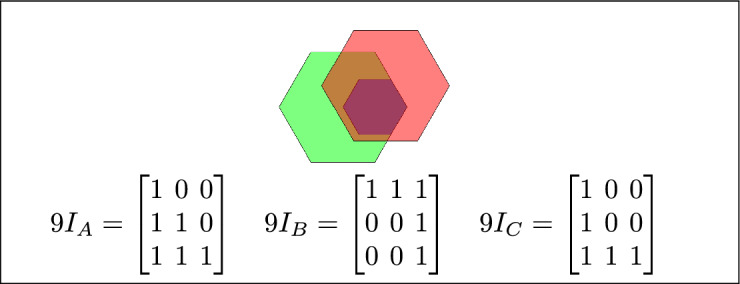
B is covered by A, C contains B, and B is inside C.

**Fig. 18:**
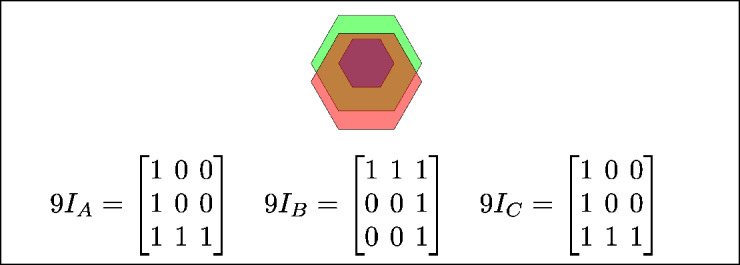
B is inside both A and C.

**Fig. 19:**
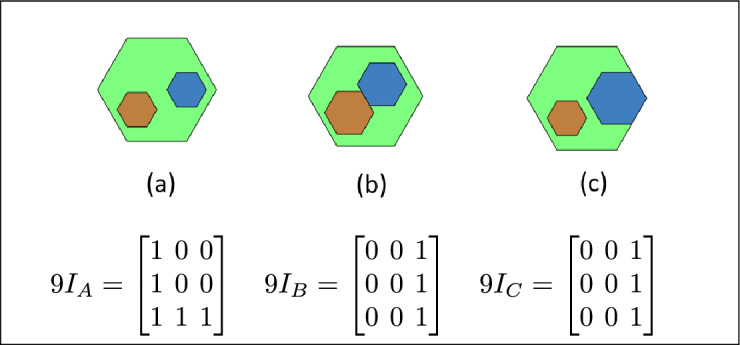
In (**a**), (**b**) and (**c**) Region A has inside relation, B and C are inner regions of A.

**Fig. 20:**
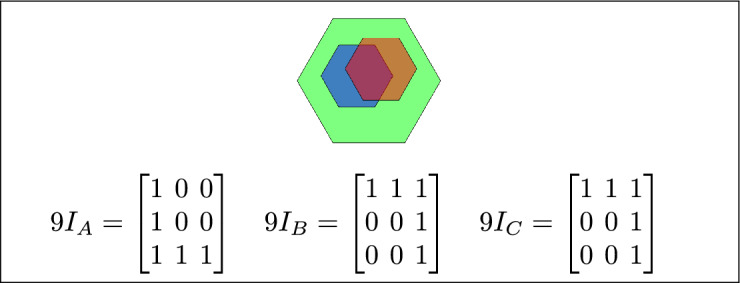
B and C are inside A and A contains B and C.

**Fig. 21:**
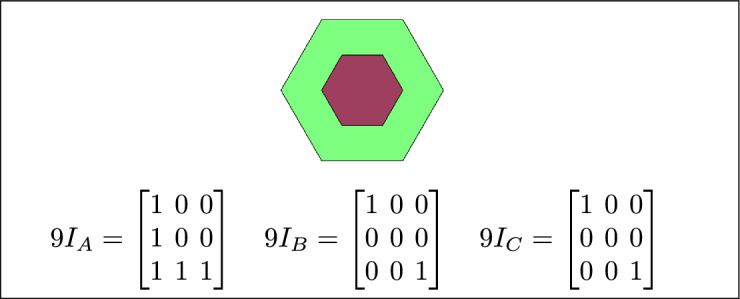
Two equal regions B and C have between relation and are inside A.

**Fig. 22:**
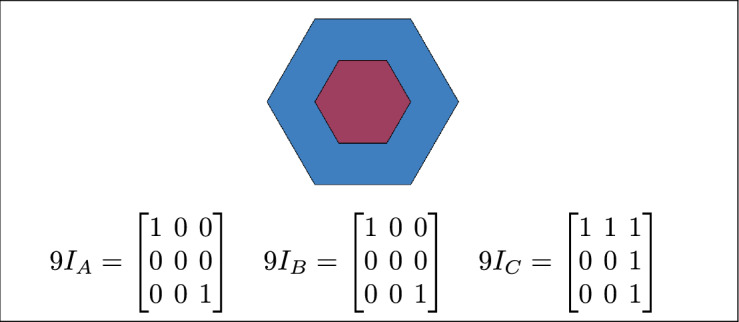
Two equal regions A and B have between relation and Region C contains A and B.

**Fig. 23:**
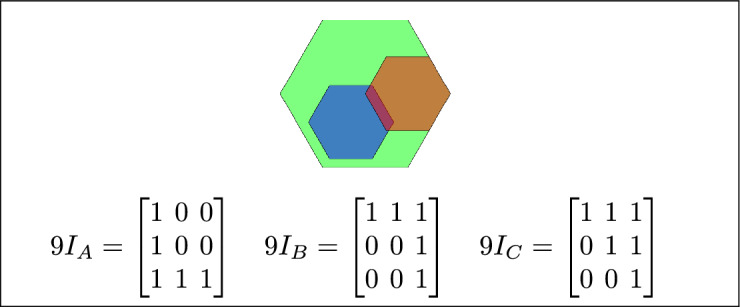
B is inside A, A contains B, and A covers C.

**Fig. 24:**
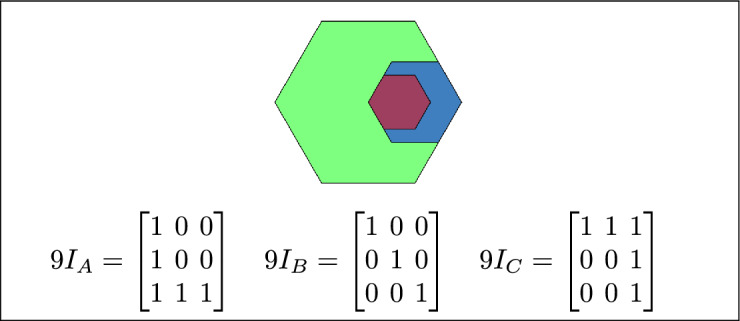
Region C is inside A, region B is in between, and A contains C.

**Fig. 25:**
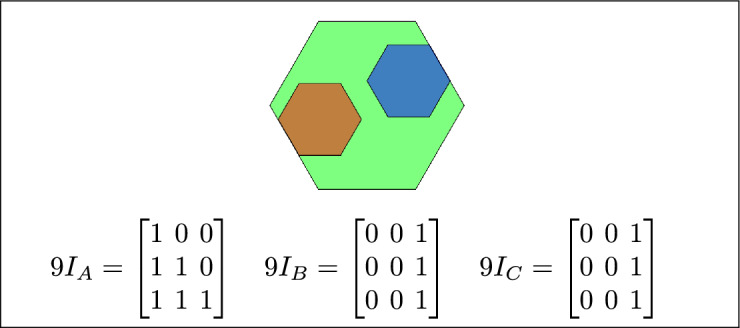
B and C are inner regions covered by A.

**Fig. 26:**
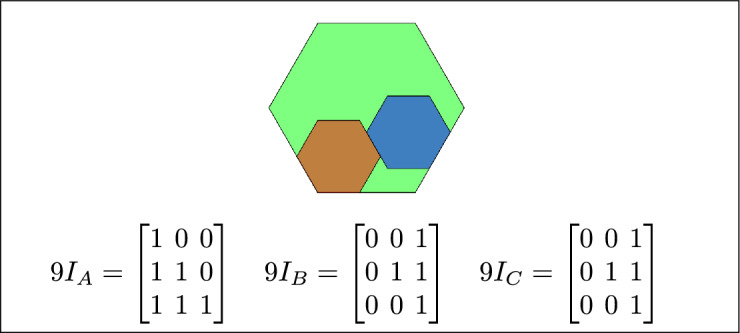
B and C are covered by A, B and C meet inside A.

**Fig. 27:**
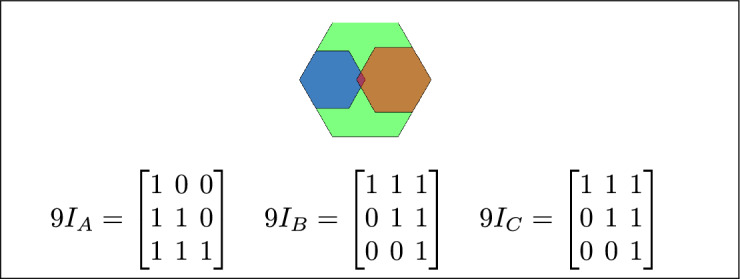
B and C are covered by A.

**Fig. 28:**
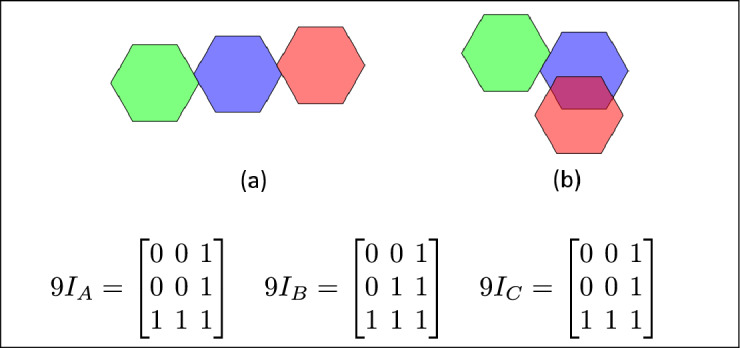
In (**a**) and (**b**) A and C are disjoint, B meets both A and C or B meet with A and overlaps with C.

**Fig. 29:**
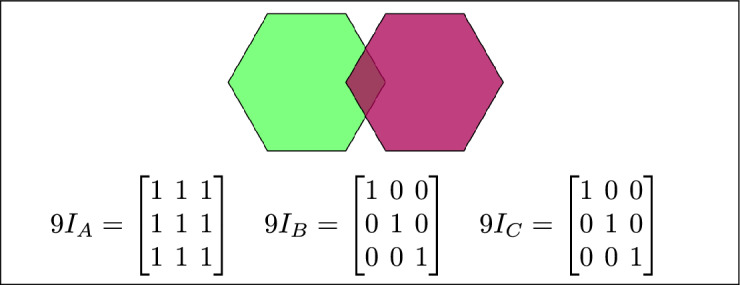
A overlaps with B and C; regions B and C are equal.

**Fig. 30:**
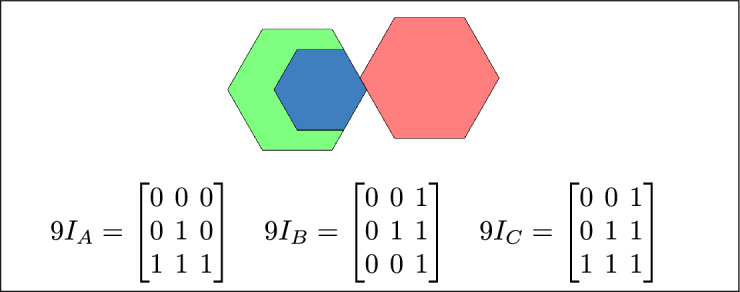
Region A meets B from the inside, B is meet-inside A, and C meets both A and B.

**Fig. 31:**
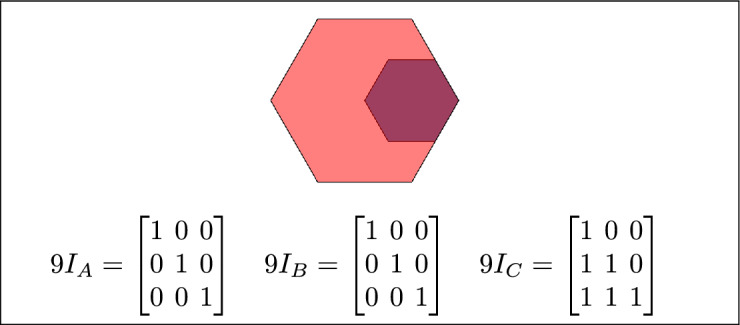
Two equal regions, A and B, are covered by C.

**Fig. 32:**
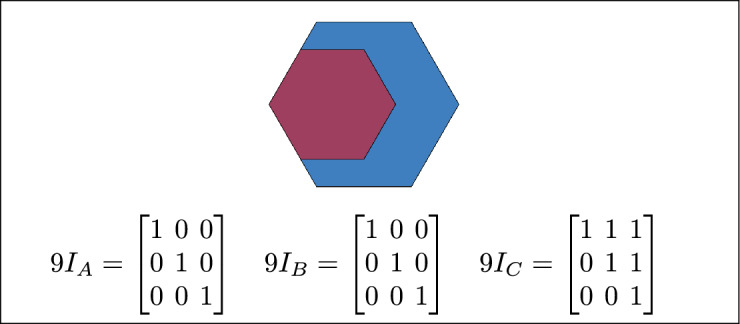
Two equal regions A and B cover C.

**Fig. 33:**
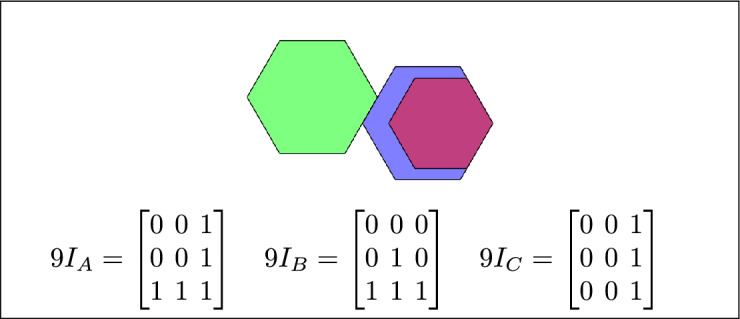
A and C are disjoint. Region C is an inner region, Region B meet C from the inside.

**Fig. 34:**
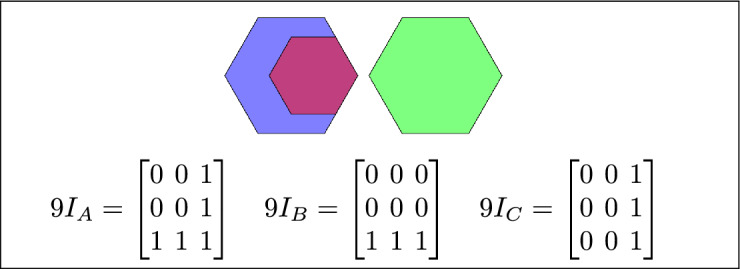
Region A is disjoint, Region B is outer, and Region C is inner.

**Fig. 35:**
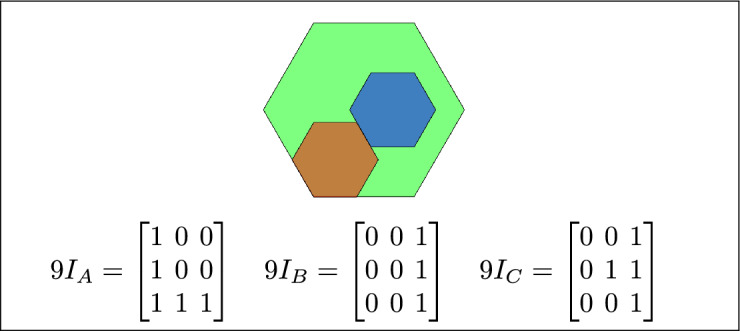
B and C are inside A, with B as an inner region and C meet A from inside.

**Fig. 36:**
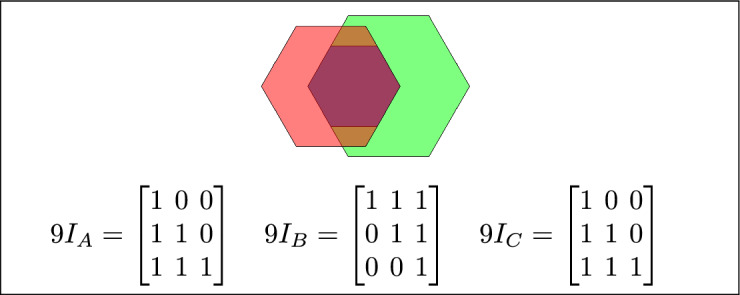
B is covered by A and C, and A and C cover B.

**Fig. 37:**
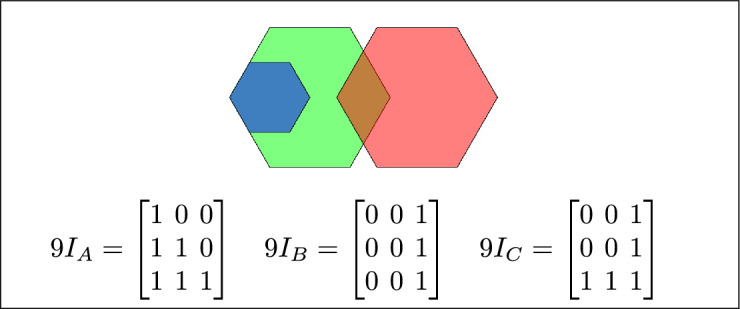
B is covered by A, region B is inner, and B is disjoint from C.

**Fig. 38:**
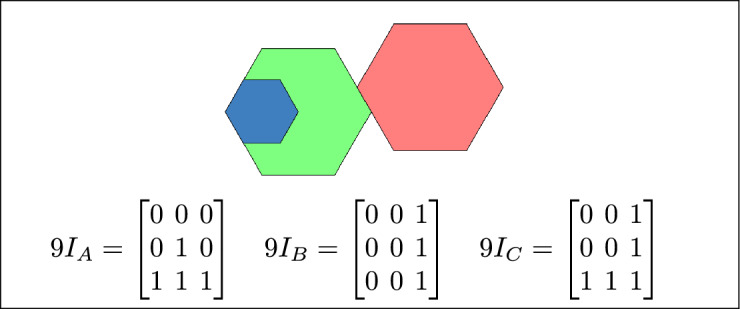
Region B is inner, meets Region A from the inside, and Region C is disjoint from B.

**Fig. 39:**
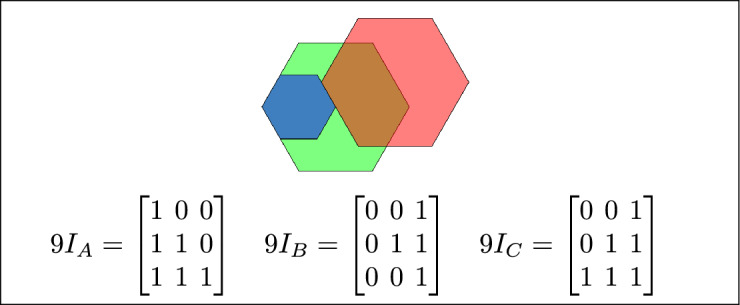
A is covered by B, region B meets A from inside, and C meets both A and B.

**Fig. 40:**
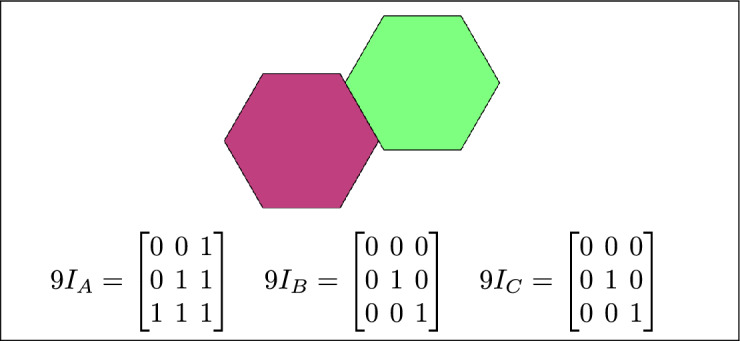
A meets two equal regions B and C; B and C have a boundary-exterior-meet.

**Fig. 41:**
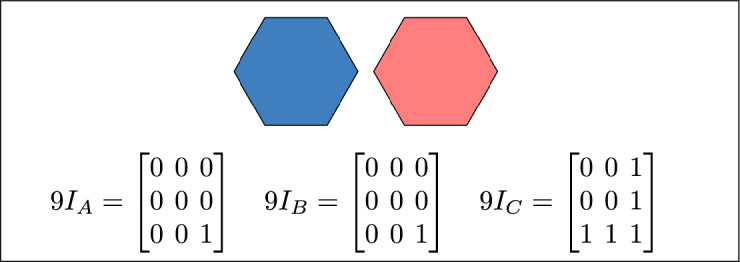
C is disjoint from two equal regions A and B, while the exteriors of A and B meet C.

The 45 specifications discussed above have three to six variants based on their positions. Change in the position of a region results corresponding change in the regions topological relation. The 16 topological relations among three simple regions, derived from 3-SRM, are listed in Table 4. where TSR, 2RD, 9*IM*, 3RD and 3SRM represents Topological spatial relations, 2 region daigram, 9 Intersection model, 3 region daigram and Three simple region model, respectively. The toplogical spatial regions that can not be detected by 2RD 9*IM* and can be detected by 3RD SRM is depicted in table 5

**Table 4:** The 16 topological relations.

TSR	2RD	9IM	3RD	3-SRM results
Disjoint		$$\begin{bmatrix} 0 & 0 & 1\\ 0 & 0 & 1\\ 1 & 1 & 1 \end{bmatrix}$$		$$9I_A = \begin{bmatrix}0 & 0 & 1 \\ 0 & 0 & 1 \\ 1 & 1 & 1\end{bmatrix}\quad 9I_B =\begin{bmatrix}0 & 0 & 1 \\ 0 & 0 & 1 \\ 1 & 1 & 1\end{bmatrix}\quad 9I_C =\begin{bmatrix} 0 & 0 & 1 \\ 0 & 0 & 1 \\ 1 & 1 & 1\end{bmatrix}$$
Meet		$$\begin{bmatrix}0 & 0 & 1 \\ 0 & 1 & 1 \\ 1 & 1 & 1 \end{bmatrix}$$		$$9I_A =\begin{bmatrix} 0 & 0 & 1 \\ 0 & 1 & 1 \\ 1 & 1 & 1 \end{bmatrix} \quad 9I_B =\begin{bmatrix} 0 & 0 & 1 \\ 0 & 1 & 1 \\ 1 & 1 & 1 \end{bmatrix} \quad 9I_C=\begin{bmatrix} 0 & 0 & 1 \\ 0 & 1 & 1 \\ 1 & 1 & 1 \end{bmatrix}$$
Overlap		$$\begin{bmatrix}1 & 1 & 1 \\ 1 & 1 & 1 \\ 1 & 1 & 1\end{bmatrix}$$		$9I_A$ = $$\begin{bmatrix}1 & 1 & 1 \\ 1 & 1 & 1 \\ 1 & 1 & 1\end{bmatrix}$$ $9I_B$ =$$\begin{bmatrix}1 & 1 & 1 \\ 1 & 1 & 1 \\ 1 & 1 & 1\end{bmatrix}$$ $9I_C$ =$$\begin{bmatrix} 1 & 1 & 1 \\ 1 & 1 & 1 \\ 1 & 1 & 1\end{bmatrix}$$
Cover		$$\begin{bmatrix}1 & 1 & 1 \\ 0 & 1 & 1 \\ 0 & 0 & 1\end{bmatrix}$$		$9I_A$ = $$\begin{bmatrix}1 & 0 & 0 \\ 1 & 1 & 0 \\ 1 & 1 & 1\end{bmatrix}$$ $9I_B$ =$$\begin{bmatrix}1 & 0 & 0 \\ 0 & 1 & 0 \\ 0 & 0 & 1\end{bmatrix}$$ $9I_C$ =$$\begin{bmatrix} 1 & 1 & 1 \\ 0 & 1 & 1 \\ 0 & 0 & 1\end{bmatrix}$$
Contain		$$\begin{bmatrix}1 & 1 & 1 \\ 0 & 0 & 1 \\ 0 & 0 & 1\end{bmatrix}$$		$9I_A$= $$\begin{bmatrix}1 & 0 & 0 \\ 1 & 0 & 0 \\ 1 & 1 & 1\end{bmatrix}$$ $9I_B$ =$$\begin{bmatrix}1 & 0 & 0 \\ 0 & 0 & 0 \\ 0 & 0 & 1\end{bmatrix}$$ $9I_C$ =$$\begin{bmatrix} 1 & 1 & 1 \\ 0 & 0 & 1 \\ 0 & 0 & 1\end{bmatrix}$$
Equal		$$\begin{bmatrix}1 & 0 & 0\\ 0 & 1 & 0 \\ 0 & 0 & 1\end{bmatrix}$$		$9I_A$ = $$\begin{bmatrix}1 & 0 & 0 \\ 0 & 1 & 0 \\ 0 & 0 & 1\end{bmatrix}$$ $9I_B$ =$$\begin{bmatrix}1 & 0 & 0 \\ 0 & 1 & 0 \\ 0 & 0 & 1\end{bmatrix}$$ $9I_C$ =$$\begin{bmatrix} 1 & 0 & 0 \\ 0 & 1 & 0 \\ 0 & 0 & 1\end{bmatrix}$$

**Table 5:** Toplogical spatial relation that can only be identified by 3 SRM.

TSR	2RD	9 IM	3RD	3-SRM results
Inner				$9I_A$ = $$\begin{bmatrix}1 & 0 & 0 \\ 1 & 0 & 0 \\ 1 & 1 & 1\end{bmatrix}$$ $9I_B$ =$$\begin{bmatrix}0 & 0 & 1 \\ 0 & 0 & 1 \\ 0 & 0 & 1\end{bmatrix}$$ $9I_C$ =$$\begin{bmatrix} 0 & 0 & 1 \\ 0 & 0 & 1 \\ 0 & 0 & 1\end{bmatrix}$$
Inside-meet and meet inside				$9I_A$=$$\begin{bmatrix} 0 & 0 & 0 \\ 0 & 1 & 0 \\ 1 & 1 & 1 \end{bmatrix}$$ $9I_B$=$$\begin{bmatrix} 0 & 0 & 1 \\ 0 & 1 & 1 \\ 0 & 0 & 1 \end{bmatrix}$$ $9I_C$=$$\begin{bmatrix} 0 & 0 & 1 \\ 0 & 1 & 1\\ 1 & 1 & 1 \end{bmatrix}$$
Outer				$9I_A$=$$\begin{bmatrix} 0 & 0 & 0 \\ 0 & 0 & 0 \\ 1 & 1 & 1 \end{bmatrix}$$ $9I_B$=$$\begin{bmatrix} 0 & 0 & 1 \\ 0 & 0 & 1 \\ 0 & 0 & 1 \end{bmatrix}$$ $9I_C$=$$\begin{bmatrix} 0 & 0 & 1 \\ 0 & 0 & 1\\ 1 & 1 & 1 \end{bmatrix}$$
Boundary-exterior-meet				$9I_A$=$$\begin{bmatrix} 0 & 0 & 1 \\ 0 & 1 & 1 \\ 1 & 1 & 1 \end{bmatrix}$$ $9I_B$=$$\begin{bmatrix} 0 & 0 & 0 \\ 0 & 1 & 0 \\ 0 & 0 & 1 \end{bmatrix}$$ $9I_C$=$$\begin{bmatrix} 0 & 0 & 0 \\ 0 & 1 & 0 \\ 0 & 0 & 1 \end{bmatrix}$$
Exterior-meet				$9I_A$=$$\begin{bmatrix} 0 & 0 & 0 \\ 0 & 0 & 0 \\ 0 & 0 & 1 \end{bmatrix}$$ $9I_B$=$$\begin{bmatrix} 0 & 0 & 0 \\ 0 & 0 & 0 \\ 0 & 0 & 1 \end{bmatrix}$$ $9I_C$=$$\begin{bmatrix} 0 & 0 & 1 \\ 0 & 0 & 1 \\ 1 & 1 & 1 \end{bmatrix}$$
Inside meet				$9I_A$=$$\begin{bmatrix} 1 & 0 & 0 \\ 1 & 1 & 0 \\ 1 & 1 & 1 \end{bmatrix}$$ $9I_B$=$$\begin{bmatrix} 0 & 0 & 1 \\ 0 & 1 & 1 \\ 0 & 0 & 1 \end{bmatrix}$$ $9I_C$=$$\begin{bmatrix} 0 & 0 & 1 \\ 0 & 1 & 1 \\ 0 & 0 & 1 \end{bmatrix}$$

## Implementation of the Robust 3-SRM model for GIS

The implementation of the Robust 3-SRM framework was carefully designed with an emphasis on robustness, reproducibility, and error-resilience. The algorithm was written in Python within the QGIS environment, leveraging the QgsGeometry and related libraries for advanced spatial operations. Unlike traditional scripts that directly manipulate geometry objects and are prone to runtime crashes, this implementation prioritizes safety checks, explicit transformations, and geometry repair mechanisms to ensure consistent outcomes, even in the presence of imperfect or corrupted data.

### Design philosophy and safety enhancements

At the core of the design is the recognition that raw geographic datasets often contain inconsistencies such as null geometries, invalid polygons, or mixed coordinate reference systems. To mitigate these, the script employs explicit safety wrappers. Instead of relying on implicit truth checks, each geometry is validated through a dedicated function that confirms non-emptiness and structural validity. This prevents the common “isEmpty” crash that arises from boolean evaluations of geometry objects. A major highlight is the transform-first approach. Before any spatial computation, features are re-projected into a target coordinate system optimized for area-based calculations (EPSG:8857). By applying transformation at the earliest stage, subsequent operations such as buffering, intersections, and unions are numerically stable and area values are expressed in meaningful metric units.

### Geometry repair and normalization

The implementation introduces multiple layers of geometry repair routines. Invalid or self-intersecting polygons are corrected using light sanitization methods, buffer operations with zero distance, or polygonization of boundary lines. If these fail, the script breaks geometries into parts, repairs them individually, and reconstructs a valid union. This staged recovery mechanism ensures that no geometry is discarded outright, preserving data integrity. To enhance matching of region names, a string normalization process is also embedded. Names of states, districts, or taluks are converted to lowercase, diacritical marks are removed, and non-alphanumeric characters are stripped. This improves the accuracy of fuzzy string matching, enabling the tool to tolerate spelling differences or formatting variations across datasets.

### Construction of 9-intersection matrices

Once the geometries for the three chosen regions (A, B, and C) are identified and repaired, the script computes their spatial relationship using the 9-Intersection Model (9-IM). For each geometry, three mutually exclusive sets are constructed:

1. The interior, approximated by shrinking the polygon slightly to exclude the boundary;
2. The boundary, represented as a stable ring created by subtracting an inner buffer from an outer buffer;
3. The exterior, defined as the trimmed universe minus the polygon with a small padding to avoid numerical overlap.

The universe itself is deliberately designed as an oversized bounding rectangle surrounding all geometries, buffered by several hundred kilometers. This ensures that exteriors are well-defined and not inadvertently clipped at the map edges. These sets are combined to generate three 3$\times$3 matrices–one for each geometry compared against the union of the other two. Each matrix entry is binary, determined by whether the intersected area exceeds a minimum threshold (e.g., 0.1 km$^{2}$). This threshold avoids spurious classifications caused by microscopic slivers or rounding errors.

###  Classification of spatial relations

The three matrices ($9I_A$, $9I_B$, and $9I_C$) are then compared against a library of predefined patterns that correspond to intuitive spatial relationships such as Disjoint, Meet, Equal, Contain, Overlap, and more nuanced cases like Boundary-Exterior-Meet. The use of patterns enables a human-readable interpretation of geometric configurations, bridging raw numerical outputs with categorical labels useful for spatial reasoning. For instance, if the interior of A intersects with the exteriors of both B and C while boundaries remain separate, the model classifies this as “Disjoint.” Conversely, if interiors overlap with substantial area and boundaries coincide, the relation may be classified as “Equal” or “Overlap.” This structured classification provides consistency across diverse datasets.

###  Quality assurance and visualization

An optional component of the implementation allows for the creation of in-memory visualization layers. These layers highlight intersections. By adding them to the QGIS project, analysts can visually confirm that the computed relations match the actual geographic configuration. This serves as a powerful tool for both debugging and validation in research contexts.

###  Human-centric robustness

The final script emphasizes usability for real-world researchers. Field detection is automatic: the program inspects the dataset and selects the most appropriate name or identifier field using a hierarchy of preferences. If a state or district name cannot be located exactly, the system offers fuzzy matches, prompting users with “Did you mean $\ldots$?” suggestions. Such design choices reduce the dependency on perfect input and align the tool with the messy nature of administrative datasets in practice.

### Outcome and results

By combining cautious geometry handling, rigorous repair methods, robust $9-IM$ construction, and a flexible classification engine, the Robust 3-SRM implementation achieves high reliability. It not only prevents common errors but also guarantees reproducibility of results across runs and datasets. The method produces transparent outputs, interpretable classifications, and optional visualizations, making it suitable for both academic research and applied spatial analysis.

**Fig. 42:**
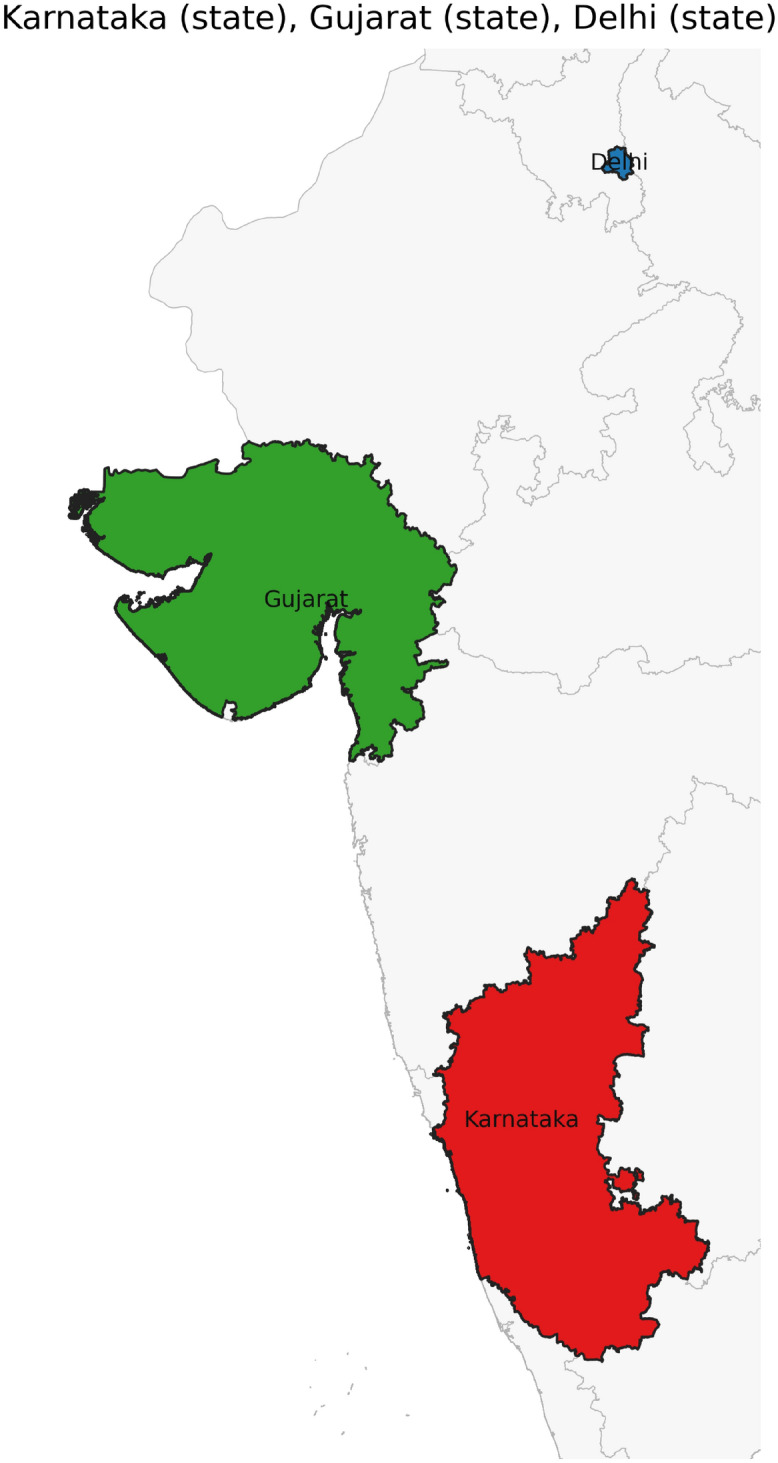
The states Karnataka, Gujarat, and Delhi have disjoint relations.

**Fig. 43:**
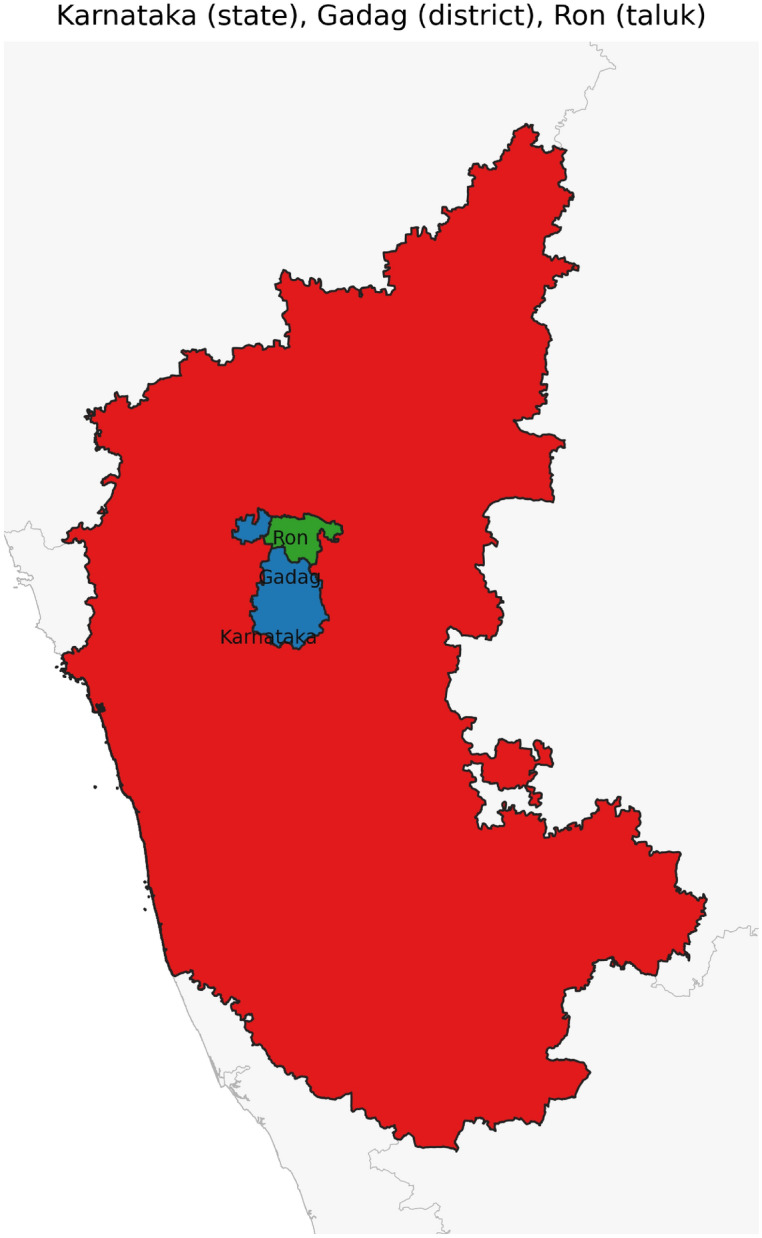
The state Karnataka, District Gadag and Taluk Ron have Coveredby-Inbetween-Covers relations.

**Fig. 44:**
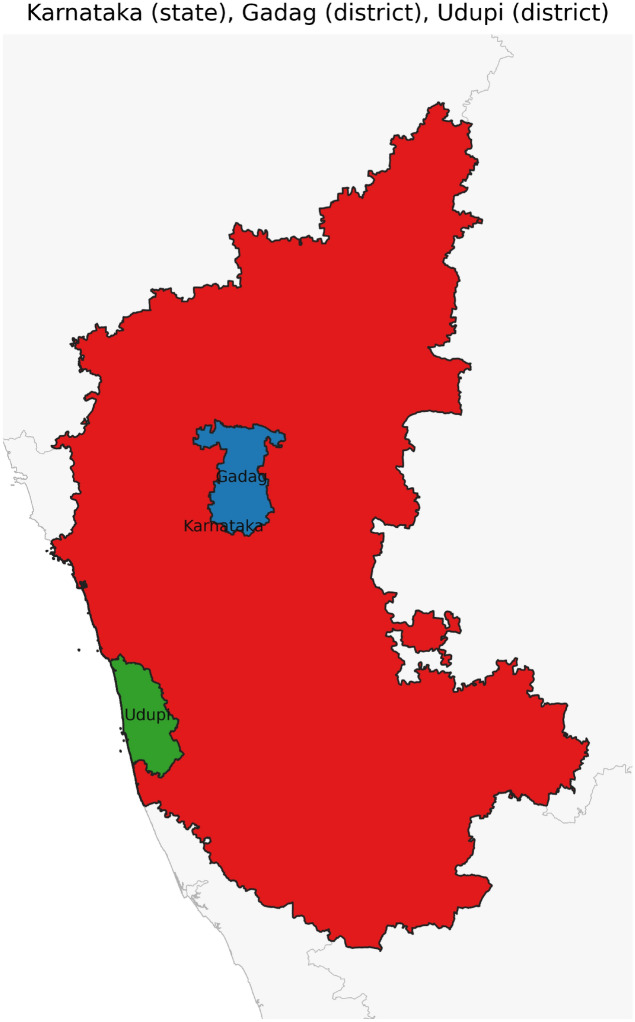
The state Karnataka, District Gadag and Udupi have Coveredby-Inner relations.

**Fig. 45:**
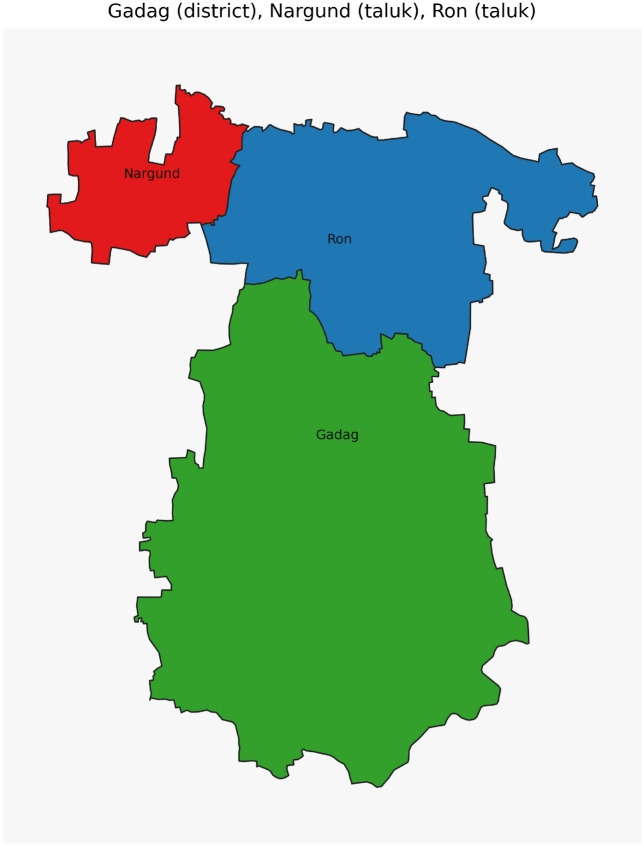
The District Gadag and Taluks Ron, Nargund have Inside-Inner-Inner relations.

**Fig. 46:**
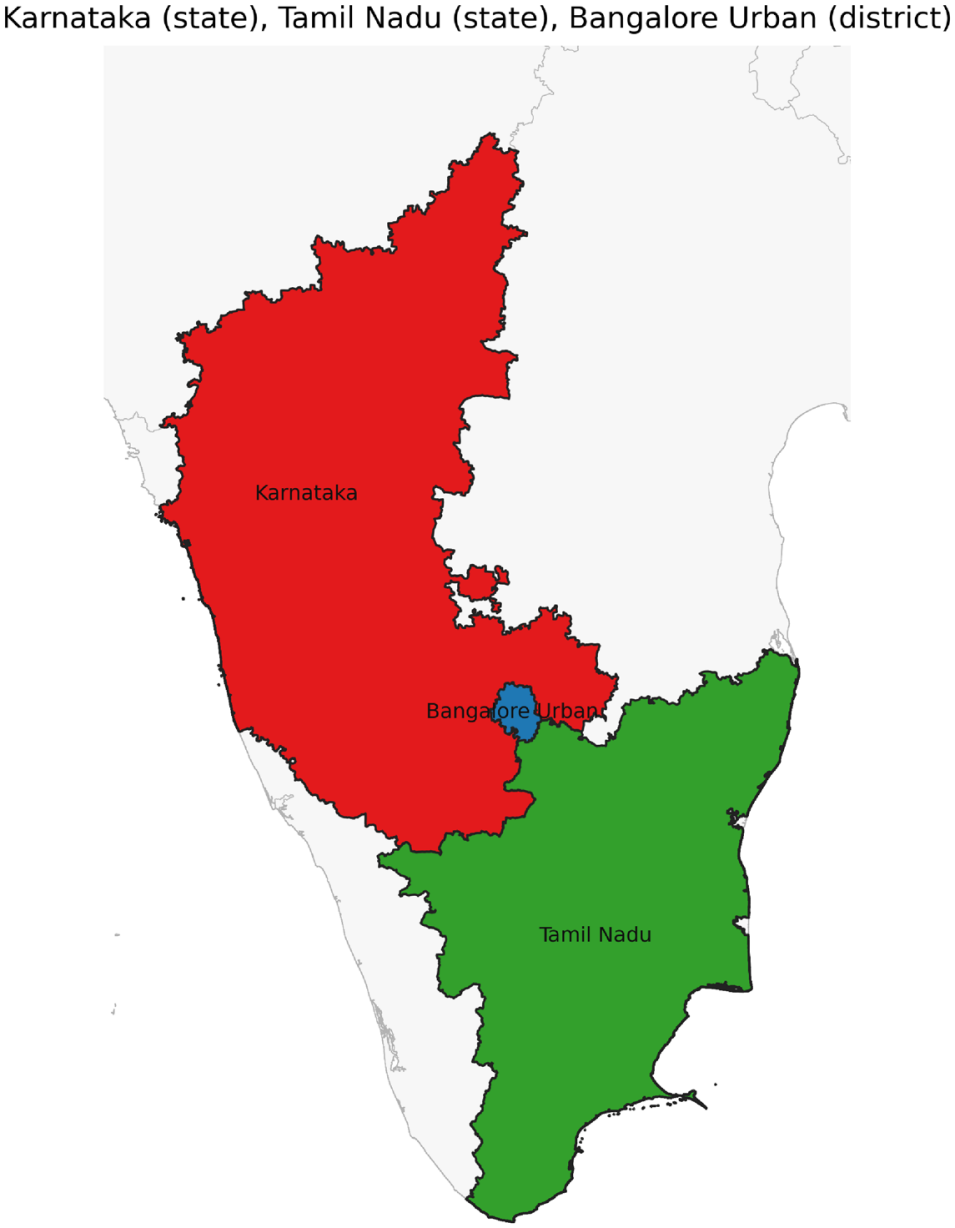
The states Karnataka, Tamil Nadu, and District Bengaluru Urban have Inside-Meet-and-Meet-Inside relations.

**Fig. 47:**
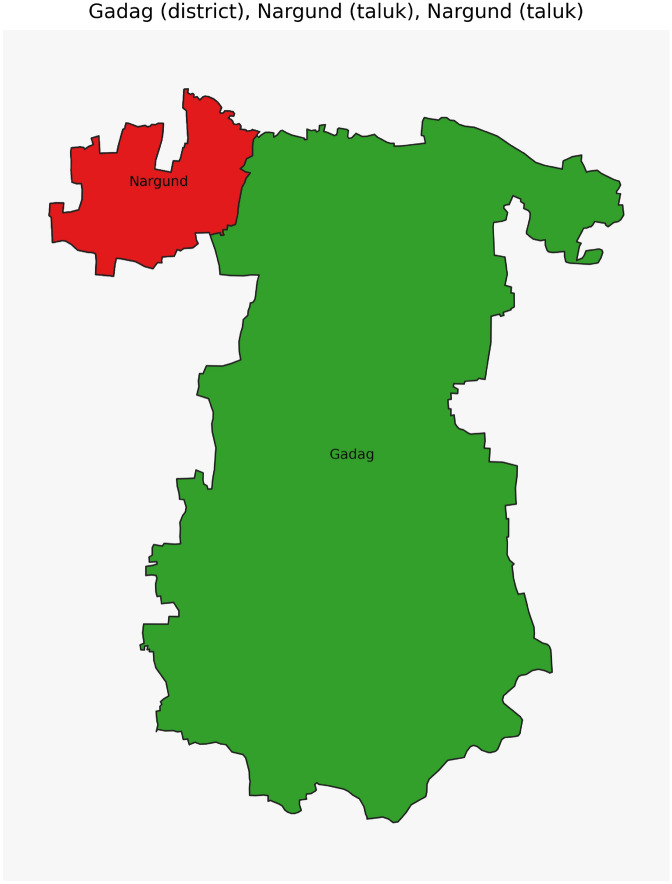
The District Gadag and Taluk Nargund, Nargund have Meet-Inside relation.

**Fig. 48:**
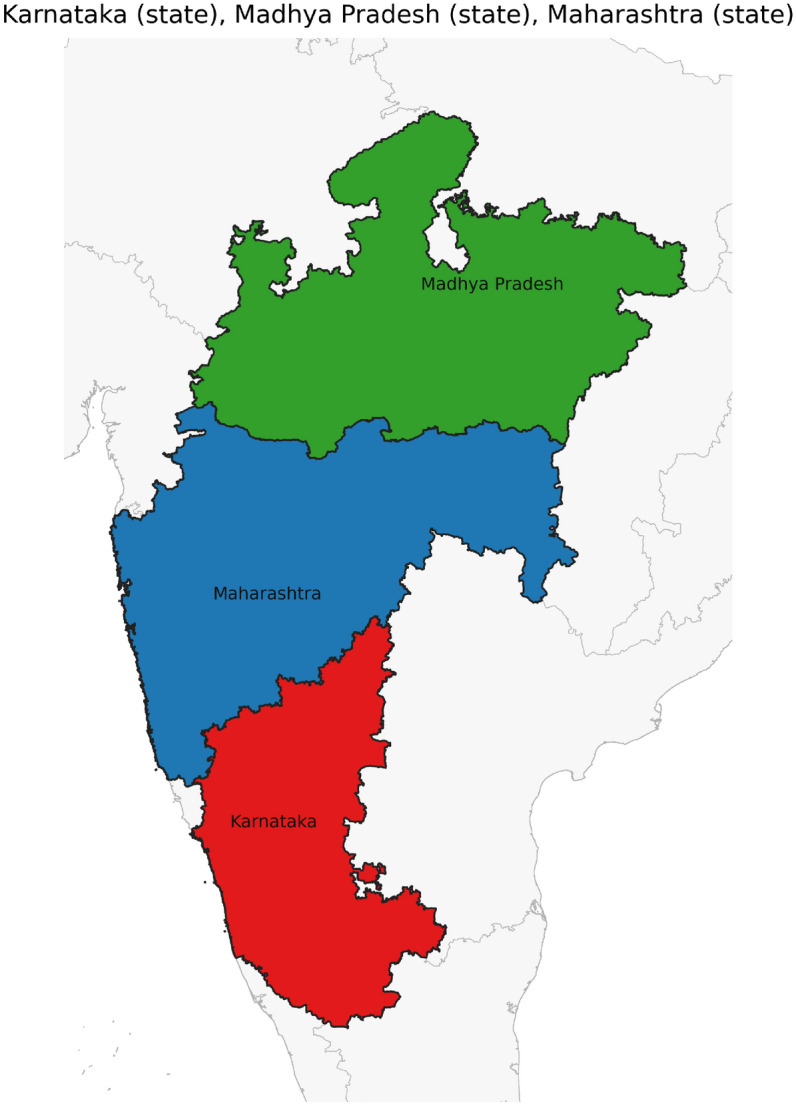
The states Karnataka, Maharastra, and Madhya Pradesh have Meet-Disjoint relations.

**Fig. 49:**
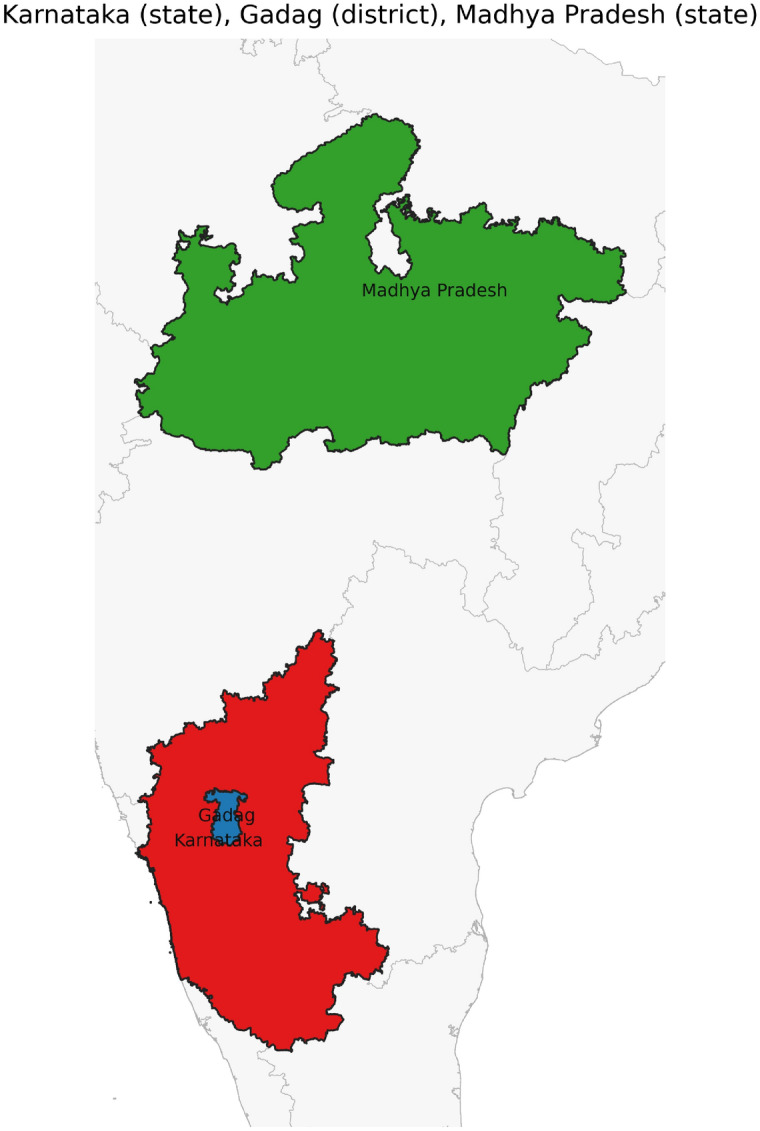
The states Karnataka, Madhya Pradesh and District Gadag have Outer-Disjoint-Inner relations.

Figure 42 has disjoint relation. Figure 43 depicts Coveredby-Inbetween-Covers relation between Karnataka state, Gadag district and Ron taluk. The covered by inner relationship is depicted in Fig. 44. The Gadag district, taluks Ron and Naragund have Inside Inner Inner relations as shown in Fig. 45. Figure 46 represents a inside-meet and meet-inside relation. The meet-inside relation is represented as in Fig. 47. Figure 48 depicts meet-inbetween relationship. Finally, the outer relationship is depicted in Fig. 49.

### Quantitative validation using official government ground truth

To complement the qualitative case studies, we performed a quantitative evaluation of the proposed 3-SRM framework. For ground truth, we used the official administrative hierarchy published by the Government of India, which specifies the authoritative parent–child relationships between States, Districts, Taluks/Sub-Districts, and Villages. These datasets (e.g., Survey of India/Census GIS layers) constitute the statutory definition of territorial boundaries and therefore provide a reliable reference for evaluation.

A representative sample of 450 spatial unit pairs across multiple administrative levels was used to compare the 3-SRM classifications with the official government-defined relations. The evaluation revealed a perfect match (100% agreement) between the 3-SRM output and the ground truth relationships.

The complete agreement between the proposed 3-SRM classifications and the administrative hierarchy defined by the Government of India demonstrates the framework’s robustness and accuracy. This result confirms that the 3-SRM method accurately captures the containment, adjacency, and boundary interactions inherent in hierarchical administrative datasets.

### Insights of 3-SRM

1. The model provides information about the topological relations that spatial regions exhibit through $9I_A$, $9I_B$, and $9I_C$
2. The 3-SRM provides a proper justification for the “overlap’ relation if one region shares its area with the remaining two regions. However, this is not the case in the following : 5.12, 5.13, 5.26, 5.35, 5.37.
3. If all three regions, or any two of them, are “disjoint”, then the 3-SRM exhibits a disjoint relation in all three components.
4. The relation “equal” holds if: (a) all three regions are equal, or (b) if two regions are equal and the third region overlaps, covers, or is covered by them. The same does not hold in the following cases: 5.38 and 5.39
5. The “meet” relation holds only if: (a) all three regions intersects in their boundary or (b) one region intersects its boundary with the boundaries of other two regions. This condition does not hold in the following cases: 5.14 and 5.17.

### Limitations of the 3-SRM for regions with holes and complex regions

When applied to regions containing holes or exhibiting complex topologies, the model faces additional challenges. The current 27-relation framework assumes regular closed, simply connected regions and therefore does not explicitly distinguish interactions involving outer boundaries versus hole boundaries. As a result, different configurations such as a region lying inside the outer shell versus lying inside an interior cavity may yield identical intersection patterns and become indistinguishable within the model. Moreover, regions with multiple holes or fractal-like boundaries produce multiple nested boundary components, which the present triadic intersection scheme cannot enumerate or encode without substantial extension of the underlying algebra. This leads to both loss of expressive power (inability to represent hole-specific relationships) and ambiguities in interpretation, especially in scenes where several boundary components interact simultaneously. Finally, reasoning with complex regions increases the potential for geometrically inconsistent or non-realizable intersection patterns, requiring additional constraints or a more sophisticated topology to ensure valid representations.

## Conclusions

This model is developed for simple spatial regions and does not address regions with internal holes or complex topologies. The topological relation among three simple spatial regions is defined using $9I_A$, $9I_B$, and $9I_C$, collectively referred to as the Three Simple Region Model (3-SRM). There exist 1,536 binary configurations in total based on the empty and non-empty intersections of the interior, boundary, and exterior of the three regions, 243 distinct intersection arrangements identified, 81 correspond to existing (non-empty) intersections (relations), from which 27 are selected to form the initial version of the 3-SRM.

Interchanging the brackets in each component of $9I_A$, $9I_B$, and $9I_C$ yields two additional models with distinct topological representations. This work examines only the first version of 3-SRM model, which identifies 16 topological relations among three simple regions in 2D space. The 3-SRM framework enhances spatial database and GIS systems, with broader applicability in cognitive science, linguistics, psychology, and computer science.”

## Future work

A comprehensive investigation of Models 2 and 3 of the 3-SRM is undertaken to systematically analyze the types of topological relations that can occur among three simple spatial regions. The study is further extended to encompass more complex configurations, including regions with internal holes, non-simple regions, simple and complex lines, as well as combinations of regions and lines, thereby broadening the scope of the model. In addition, practical implementation of the 3-SRM models within GIS environments is explored, providing a concrete platform for applying the framework. These extensions and implementations underscore the model’s potential for broader applicability across diverse domains, including cognitive science, linguistics, psychology, and computer science, where understanding multi-object spatial relations is critical.

## Data Availability

All data generated or analysed during this study are included in this published article
